# Fluoro-Functionalized Silsesquioxane Polymer-Based High Hydrophobic Coatings for Enhancing Properties of Kraft Paper

**DOI:** 10.3390/ijms262311719

**Published:** 2025-12-03

**Authors:** Mark A. Stepanov, Yuliya I. Bolgova, Olga M. Trofimova, Alexander S. Pozdnyakov

**Affiliations:** A.E. Favorsky Irkutsk Institute of Chemistry of the Siberian Branch of the Russian Academy of Sciences, 1 Favorsky Str., 664033 Irkutsk, Russia; stepanov@irioch.irk.ru (M.A.S.); omtrof1@irioch.irk.ru (Y.I.B.); omtrof@irioch.irk.ru (O.M.T.)

**Keywords:** polyfluorinated silsesquioxane, kraft paper, dip coating, high hydrophobicity, mechanical strength, moisture resistance

## Abstract

Paper plays an important role in the packaging industry due to its low cost, light weight, recyclability and biodegradability. However, the use of paper as a packaging material is severely limited due to its hydrophilicity caused by the hydroxyl groups of cellulose. This study reports a simple preparation of highly hydrophobic kraft paper by a one-step dip coating method using [3-(2,2,3,3-tetrafluoropropoxy)propyl]silsesquioxane, {3-[(2,2,3,3,4,4,5,5-octafluoropentyl)oxy]propyl}silsesquioxane or {3-[(2,2,3,3,4,4,5,5,6,6,7,7-dodecafluoroheptyl)oxy]propyl}silsesquioxane as hydrophobic agents. As a result of modification of kraft paper, a stable covalently bonded coating is formed on its surface. The coated kraft paper has demonstrated (1) high water resistance (the water contact angle (WCA) values were 124–141°, and the water absorption and the water vapor permeability (WVP) rates were significantly decreased), (2) excellent resistance to aggressive environments and temperature, (3) enhanced mechanical properties (tensile strength increased from 46.8 to 70.8 MPa), and (4) high wear resistance, as confirmed by sandpaper abrasion, bending, and finger-wipe tests. It was shown that the maximum contact angle values were achieved for kraft paper modified with a 5% polymer solution. The results of this study have great potential, given the simplicity of the modification method, for use in the production of paper-based packaging materials with water-repellent, enhanced mechanical and moisture-protective properties.

## 1. Introduction

Currently, there is an increasing interest in the development of hydrophobic and oil-resistant materials, which are becoming increasingly in demand in various industries. These materials have high performance characteristics, which makes them especially valuable for use in the packaging, textile, and construction industries. Among the diverse classes of high-performance materials, the ubiquitous use of plastic whose chemical composition is based on petroleum products leads to the accumulation of plastic waste in the environment [1,2]. Global plastic production reached 407 million tons per year [3,4], of which about 38% is used in the production of packaging materials. At the same time, about 244 million tons of plastic is thrown into landfills and ends up in the ocean every year. Plastic pollution poses a danger to drinking water, food, soil, aquatic life and the ecosystem as a whole [3,5,6]. To solve this problem, more environmentally friendly alternatives are highly desirable, among which special attention is paid to the creation of biodegradable plastics and paper-based packaging materials.

Paper, cardboard, corrugated cardboard, etc., are widely used materials for food packaging due to their numerous advantages such as low cost, good mechanical properties, recyclability, light weight and biodegradability [7,8,9,10,11]. Paper materials are made from wood, the main component of which is fibrous cellulose. Since cellulose is a structural polysaccharide with many hydroxyl groups, paper has hydrophilicity, which leads to high water absorption [12,13]. Thus, unmodified cellulosic materials do not have the water- and oil-repellent properties required for a wide range of applications. As a result of polar interactions and the formation of hydrogen bonds between water molecules and hydroxyl groups of cellulose, the contact angle of unmodified cellulose materials can vary from 10° to 50° even on absolutely smooth surfaces of pure cellulose, depending on the degree of its crystallinity. These contact angle values are significantly below the hydrophobicity threshold, which is usually assumed to be 90° [14]. Natural cellulose products contain greater or lesser amounts of hemicelluloses and lignin. These substances are even more hydrophilic than cellulose because of the numerous phenolic and acetal groups present in their molecular structures [15]. The absence of repellent properties against water and steam is also due to the porous structure of cellulose fibers. With pore sizes of 100–200 µm, they have a strong capillary effect, which is additionally enhanced by their inner surface’s low contact angle. As a result, water trapped on the surface of unmodified paper is quickly absorbed into its inner surface, and water vapor, having penetrated the pores, lingers on their inner walls, where it then diffuses into the fibers [16]. Thus, the porous geometry and polar chemical composition make the paper unsuitable for use in areas involving direct contact with liquid [17].

To achieve hydrophobicity in materials, a necessary condition is a low surface energy at the interface. This parameter is widely used in the creation of anti-fouling, biocompatible, water- and oil-resistant, and dirt-resistant paper-based packaging materials. Currently, various polymer compounds are used to achieve low free surface energy on substrates. Among them, both fluorine-containing and non-fluorinated polysiloxanes, polyesters, polyacrylates, etc., can be distinguished.

Organosilicon compounds are widely used for hydrophobization of surfaces of various chemical natures. The combination of fluorine and organosilicon chemistry makes it possible to form coatings with a fairly low surface energy, highly hydro- and oleophobic properties, good chemical adhesion to surfaces, and excellent heat- and frost-resistant properties, which makes functional fluorinated organosilicon compounds the most popular and widely used in practice. Both low-molecular-weight and high-molecular-weight organosilicon compounds are used [18,19,20].

Both physical and chemical modification methods are used to create hydrophobic paper. SiO_2_ nanoparticles in combination with various chemical agents are used to impart hydrophobic properties to paper. For example, Ogihara et al. [21] describes a simple spray coating technique that is widely used in industry. The use of an alcohol suspension of SiO_2_ nanoparticles of different sizes made it possible to achieve WCA from 126° to 155°. A one-step approach to the creation of superhydrophobic paper by introducing fluorinated SiO_2_ into a cellulose matrix by spraying was demonstrated in the study [22]. At the same time, the coatings proved to be extremely repellent against water (WCA 176°) and organic solvents (WCA > 130°), with a surface tension of about 45 mN/m and a slip angle values below 5°. In addition, this paper demonstrated high mechanical strength, as well as self-cleaning and anti-icing properties. Yang and Deng proposed a method for multi-layer deposition of a coating based on polydiallyldimethylammonium chloride and SiO_2_ particles, followed by surface modification with 1*H*,1*H*,2*H*,2*H*-perfluorooctyltriethoxysilane [23]. Wang et al. used a hydrolyzed mixture of fluorinated triethoxysilane and tetraethoxysilane to create a superhydrophobic surface on a wide range of substrates (fabric, paper, glass and silicon wafers) using a one-step coating method [24]. It was found that the nature of the substrate used does not affect the hydrophobic properties of the surface. Recently developed omniphobic coatings help solve the problem of creating surfaces resistant to all types of liquids. For example, a facile method for producing omniphobic paper was demonstrated using a one-step solvent-free vapor deposition–silanization process using chlorosilane molecules (1,3-dichlorotetramethyldisiloxane) to create polymer brushes on paper substrates [25]. A more innovative approach is to create mechanically strong and hydrophobic paper by combining micro–nano-fibrillated cellulose fiber and chitin nanocrystals followed by impregnation with polymethylsiloxane or trimethoxysilane embedded in the structure of cellulose paper [26,27,28,29]. Authors of other studies presented more complex approaches [30,31,32]. They developed an effective hydrophobic kraft paper coated with a mixture of zein, polyvinyl alcohol, corn starch, and a graft copolymer of polydimethylsiloxane with chitosan. The resulting kraft paper demonstrated good oil-resistant (WCA 77°) and water-repellent (WCA 119°) properties, as well as improved vapor barrier properties. Through layer-by-layer modification using chitosan followed by treatment with polydimethylsiloxane, water- and oil-repellent cardboard was successfully obtained [33].

Despite a number of achievements in the production and study of hydrophobic packaging materials, the modification of polar, highly porous cellulose substrates, such as paper, fabrics, cardboard, etc., into materials with enhanced performance characteristics and pronounced water- and oil-repellent properties remains a complex but extremely relevant and promising task [34].

This paper presents the results of a study on the modification of kraft paper, conducted with the aim of creating a highly effective environmentally friendly packaging material with pronounced hydrophobic properties and high resistance to physicochemical influences using fluorinated organosilicon polymers as hydrophobic agents. Fluorosiliconorganic polymers are a promising and dynamically developing class of heterochain high-molecular-weight compounds [35]. The choice of polymers based on organosilicon compounds with polyfluorinated substituents is due to their low surface energy, hydrophobic and oleophobic properties, high heat resistance, minimal adhesion, and high resistance to physicochemical and mechanical influences [36,37,38], as well as the absence of toxicity [39,40,41]. To obtain stable hydrophobic materials, we propose using [3-(polyfluoroalkoxy)propyl]silsesquioxane polymers with different fluoroalkyl chain lengths synthesized by us. The hydrophobicity of the modified kraft paper was determined by measuring the contact angle. The physicochemical properties of the material were characterized using FTIR, SEM, and EDS, alongside the main physical parameters such as thickness, basis weight and coating load. To evaluate the improvement in the functional properties of the obtained highly hydrophobic kraft paper, its performance characteristics were analyzed in detail, including tests for mechanical strength and wear resistance, moisture resistance, thermal stability and resistance to aggressive environments. Hydrophobically modified kraft paper has significant practical value as a universal material for various fields of industry.

## 2. Results and Discussion

### 2.1. Modifying Hydrophobic Agents’ Synthesis and Characterization

**Scheme 1:**
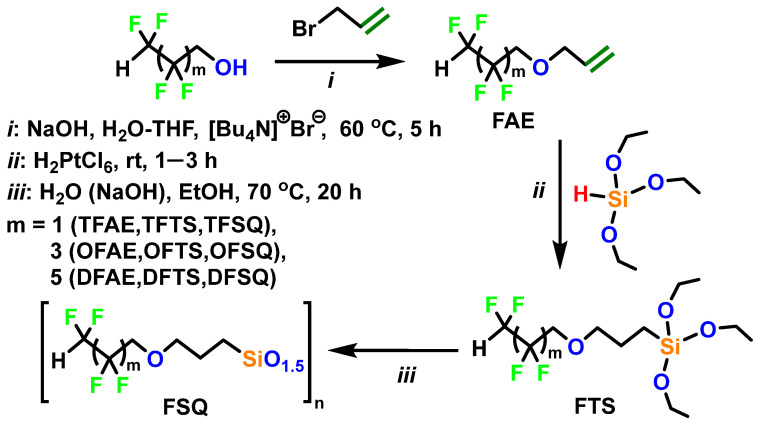
The synthetic route of FSQs.

The synthesis of polyfluorinated silsesquioxane polymers (FSQs) was carried out by the hydrolytic polycondensation of alkoxysilanes (FTSs) under basic catalysis (Scheme 1), according to the procedure described by us in [42]. For this purpose, triethoxy[3-(2,2,3,3-tetrafluoropropoxy)propyl]silane (TFTS), triethoxy{3-[(2,2,3,3,4,4,5,5-octafluoropentyl)oxy]propyl}silane (OFTS), and {3-[(2,2,3,3,4,4,5,5,6,6,7,7-dodecafluoroheptyl)oxy]propyl}(triethoxy)silane (DFTS) were used, which were obtained via two stages, as detailed in our previous study [43]. In the first stage, allyl 2,2,3,3-tetrafluoropropyl ether (TFAE), allyl 2,2,3,3,4,4,5,5-octafluoropentyl ether (OFAE), and allyl 2,2,3,3,4,4,5,5,6,6,7,7-dodecafluoroheptyl ether (DFAE) were synthesized as a result of interaction of the corresponding polyfluorinated alcohol and allyl bromide under phase transfer catalysis conditions in the presence of tetrabutylammonium bromide [Bu_4_N]^+^Br^−^ as a phase-transfer catalyst (Scheme 1). Next, hydrosilylation of the obtained fluorinated olefins by HSi(OEt)_3_ using a Pt-based homogeneous Speier catalyst resulted in polyfluorinated triethoxysilanes (FTSs) (Scheme 1). FTIR and multinuclear NMR spectra of polyfluorinated allyl ethers (FAEs) and FTSs are given in the Supplementary Materials (Figures S1–S23). It should be noted that the used initial precursors—which are products of telomerization of tetrafluoroethylene with methanol a general formula of HCF_2_(CF_2_)_m_CH_2_OH, where m denotes different mass contents of fluorine atoms—are convenient, cheap and easily accessible reagents.

The target FSQ products were soluble in DMSO, DMF, tetrahydrofuran, ethanol, and acetone, but were insoluble in water, toluene, dichloromethane, and chloroform. Good solubility will contribute to the use of these compounds as surface modifiers in various fields.

The chemical structure of FSQs has been proven by FTIR (Figure S24) and ^1^H, ^13^C,^19^F, and ^29^Si NMR (Figures S25–S36). Polyfluoroalkoxyalkyl groups remain unaffected by hydrolysis and condensation reactions. The absence of characteristic resonances of the triethoxysilane monomer at 3.81 and 1.22 ppm indicates successful hydrolysis of the Si(OEt)_3_ group. The broadened proton signals of all units indicate that the monomer was condensed into a relatively high-molecular-weight polymer. In the FTIR spectra of the FSQs, the characteristic absorption frequencies of the Si–O–C bonds of the ethoxysilyl group at 2890–2887 cm^–1^, 1120–1079 cm^–1^ and 811–793 cm^–1^ [44] are absent, which also confirms the completion of the hydrolysis reaction. The medium intensity absorption band at 937–944 cm^–1^ and a broad and weak characteristic absorption band in the region of 3300–3500 cm^–1^ correspond to residual silanol groups Si–OH. Due to the presence of reactive Si-OH groups, the coating forming as a result of substrate modification will have good adhesion due to the formation of hydrogen and/or covalent bonds with the functional groups on the surface of processed material, in particular, for paper substrates due to condensation with hydroxyl groups of cellulose.

The molecular weight characteristics of the polyfluorinated silsesquioxane homopolymers were studied by gel permeation chromatography using a tetrahydrofuran as an eluent. The results obtained are presented in Table 1, and the GPC curves are given in the Supplementary Materials (Figure S37).

From the data presented it is evident that the products have relatively high average molecular weights and, therefore, are macromolecules and not oligomeric compounds. All obtained polymers have low polydispersion (Đ), which indicates the formation of F-SQs with a relatively homogeneous size distribution.

Thermogravimetric analysis (TGA) performed in air shows that all the investigated hydrophobic agents (FSQs) remain stable up to 210 °C, after which a noticeable weight loss begins as a result of thermal oxidative degradation. Based on the data presented in Figure S38, it can be concluded that the TFSQ polymer exhibits the lowest thermal stability: its temperature of 5% weight loss (T_d5%_) is 220 °C. For OFSQ and DFSQ, T_d5%_ is slightly higher and equals 243 °C and 288 °C, respectively.

### 2.2. Modification of Kraft Paper

**Figure 1:**
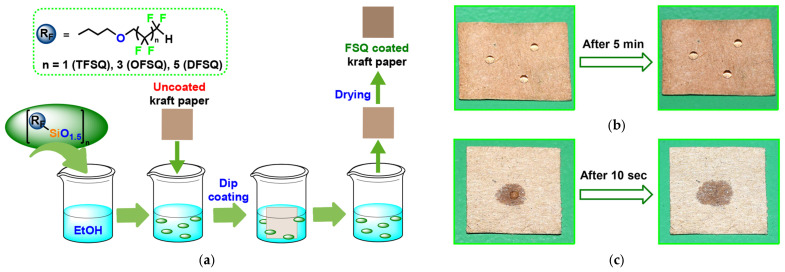
One-step dip coating process for kraft paper with FSQs (**a**); images of water droplets on the surface of OFSQ_5%_ (**b**) and uncoated kraft paper (**c**).

Chemical modification with polymers is a highly effective approach to creating materials with unique properties. Synthesized polysilsesquioxanes with polyfluorinated substituents in the side-chain FSQs were used for hydrophobization of kraft paper. Highly hydrophobic coatings were obtained by a dip coating method (Figure 1a) using ultrasound (ultrasound contributed to a better and more uniform distribution of the compounds over the surface). Figure 1b,c show an image of water droplets on OFSQ_5%_ and on an uncoated kraft paper sample, respectively. The drop shape on the OFSQ_5%_ persists for 5 min after its application, while the uncoated sample absorbs a drop of water immediately after its application, since the high hydrophilicity of cellulose paper, due to the large number of hydroxyl groups, promotes rapid wetting with water (WCA 0°).

**Scheme 2:**
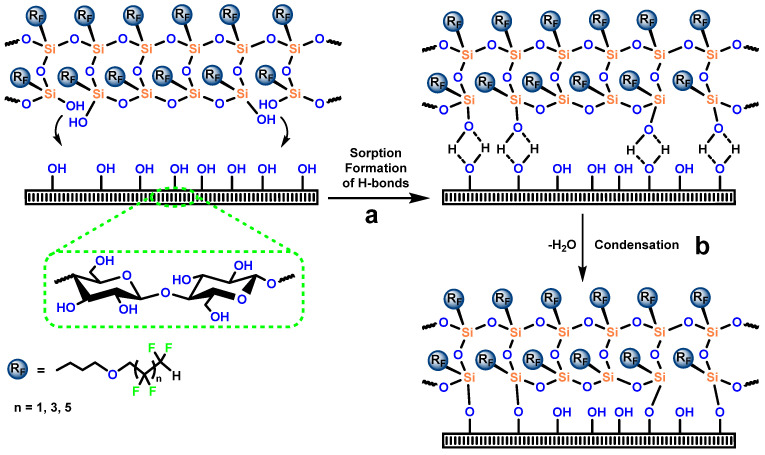
Schematic representation of the formation of a siloxane coating: (**a**) sorption of silanol groups by means of hydrogen bonds; (**b**) formation of covalent bonds due to heat treatment.

The mechanism of formation of a hydrophobic coating on kraft paper is based on the formation of covalent bonds due to the condensation reaction of residual silanol groups of the polymer with –OH groups of cellulose (Scheme 2).

At the initial stage, the residual reactive silanol groups (Si-OH) of the corresponding polymer FSQs in solution are locally fixed to the substrate mainly by hydrogen bonding, since they have a high affinity for the surface of the paper substrate [45]. At the subsequent stage, heat treatment initiates a condensation reaction, as a result of which Si-O-C interfacial bonds are formed [46,47], and the polymer chain covers a significant surface area of the substrate, which helps to block a large number of hydroxyl groups on its surface [48]. At the same time, the polyfluoroalkyl substituents are oriented in the top layer of the coating, thereby providing hydrophobic properties [48]. Thus, all these interactions lead to the formation of a stable and durable hydrophobic film, characterized by a high degree of crosslinking by covalent bonds both between molecules and with a paper substrate.

### 2.3. Highly Hydrophobic Kraft Paper Properties and Performance Tests

#### 2.3.1. Contact Angle Measurements

The water contact angle is a good indicator of the relative hydrophobicity or hydrophilicity of a sample [49]. The WCA values for coated kraft paper are presented in Table 2.

The obtained results show that modifications with all compounds used in this study cause the formation of a highly hydrophobic (WCA > 120°) surface of kraft paper. In Table 2, there is a clear correlation between the values of the contact angle measured on the surface of coated kraft paper and the length of the fluoroalkyl chain, determined by the number of CF_2_ fragments in the chemical structure of FSQs. The highest WCA values were observed for surfaces modified with fluorinated polysilsesquioxane DFSQ, which has the longest fluoroalkyl chain. It follows from this that differences in the chemical structure of compounds have a significant effect on their hydrophobic ability. Analysis of the data in Table 2 suggests that the concentration of the polymer in the solution also affects the hydrophobic properties of kraft paper. Thus, an increase in the polymer concentration to 5% improved the hydrophobic properties of the treated paper compared to TFSQ_3%_, OFSQ_3%_, and DFSQ_3%_ samples, probably due to filling a sufficiently large number of paper pores with polymer. As a result, the contact angle values increased by about 5–7°. However, with a further increase in the polymer content (samples TFSQ_7%_, OFSQ_7%_, and DFSQ_7%_), the WCA values changed little and even decreased slightly by approximately 4–6°. This is possibly due to a decrease in the degree of pore filling which can be caused by steric hindrance arising from mutual collisions and intertwining of macromolecules. Thus, the optimal polymer concentration for modifying kraft paper can be considered to be 5 wt.%, which allowed TFSQ_5%_, OFSQ_5%_, and DFSQ_5%_ samples to be selected for further study.

#### 2.3.2. Morphological and Structural Characterization

**Figure 2:**
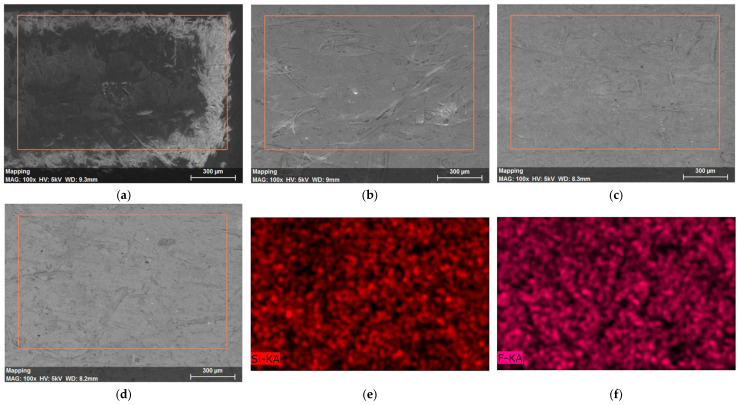
SEM images of uncoated kraft paper (**a**), TFSQ_5%_ (**b**), OFSQ_5%_ (**c**), and DFSQ_5%_ (**d**). EDS mapping of Si (**e**) and F (**f**) elements on the surface of DFSQ_5%_.

The hydrophobicity of coated kraft paper depends not only on the chemical composition of the coatings, but also on its morphological features. Therefore, the morphology of the paper surface before and after modification was studied by scanning electron microscopy (SEM) (Figure 2).

Micrograph 2a shows that the uncoated kraft paper substrate consists of interwoven cellulose fibers that form a porous structure and provide the paper with its initial roughness, which explains why water droplets easily pass through the cellulose matrix of the paper [50]. In Figure 2b–d, it can be observed that the surface of the modified paper samples has become smoother and more uniform compared to clean paper. This phenomenon can be explained by the fact that polysilsesquioxane macromolecules penetrated into the pores formed by cellulose fibers and fixed on the cellulose through Si-O-C covalent bonds, thereby filling and masking these pores. This makes the paper impervious to water droplets, which is in good agreement with the measurement results of the WCA values (Table 2).

In addition, elemental mapping analysis using energy-dispersive X-ray spectroscopy (EDS) was carried out for the surface of the DFSQ_5%_ (Figure 2e,f), TFSQ_5%_, and OFSQ_5%_ (Figure S39a,b). The mapping images of silicon and fluorine atoms confirm the homogeneous and even distribution of these elements on the substrate surface. This indicates that the polymer macromolecules have successfully fixed on the substrate. Based on the results obtained using SEM and EDS, it can be concluded that the modification of the paper substrate was successfully carried out, as well as the formation of a favorable morphology necessary to impart highly hydrophobic properties to kraft paper.

**Figure 3:**
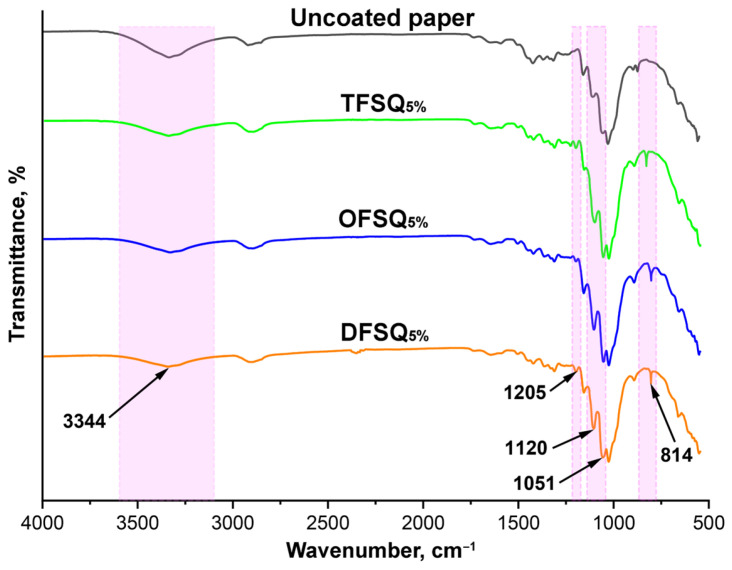
FTIR spectra of TFSQ_5%_, OFSQ_5%_, DFSQ_5%_, and uncoated kraft paper.

The obtained highly hydrophobic kraft paper was characterized by FTIR spectroscopy (Figure 3) in order to confirm the successful hydrophobization of kraft paper by FSQs, as well as to establish their binding to the surface of kraft paper.

The characteristic broad absorption band with a maximum at 3344 cm^−1^ corresponds to stretching vibrations of –OH groups in cellulose [51]. The absorption frequency at 2921 cm^−1^ represents the asymmetric stretching vibration of C-H bonds (sp^3^), and the absorption bands at 1032 and 1051 cm^−1^ correspond to the stretching vibration of C-O bonds in uncoated kraft paper [51]. In the FTIR spectra of the obtained hydrophobic kraft paper, new absorption bands appeared at 1205 and 814 cm^−1^, corresponding to the stretching vibrations of the C-F bond [44,52,53], present in polysilsesquioxanes, and the stretching of the Si-O-C bond [44,54], respectively. The signal at 814 cm^−1^ indicates the formation of a Si-O-C interfacial covalent bond through condensation between the silanol Si-OH groups of polysilsesquioxane and the hydroxyl groups of cellulose. In addition, the intensity of the absorption band of hydroxyl groups at 3344 cm^−1^ in the FTIR spectra of the coated kraft paper decreased as a result of the formation of Si-O-C bonds through a condensation reaction [29]. This indicates successful covalent binding of polysilsesquioxane to cellulose paper during its hydrophobization. The absorption band at 1032 cm^−1^ corresponds to the stretching of the C-O bond of the C-O-C group [55] located in the polyfluorinated substituent of polysilsesquioxane and overlaps with the absorption band of the C-O bond in cellulose. The increase in the intensity of the absorption bands at 1120 and 1051 cm^−1^ indicates the presence of stretching asymmetric and symmetric vibrations of the siloxane bonds Si-O-Si [56], overlapping with the vibrations of the C-O bond of cellulose. Thus, the analysis of the FTIR spectra of the obtained hydrophobic kraft paper indicates the successful covalent fixation of polysilsesquioxane on kraft paper during its hydrophobization.

#### 2.3.3. Thickness, Basis Weight, and Coating Load

The basis weight and thickness of the paper are important parameters that have a significant impact on the mechanical properties of the material [57]. Therefore, to assess the hydrophobic coating content, the above parameters were determined for both coated and uncoated kraft paper and are presented in Table 3.

The thickness of uncoated kraft paper was 185 μm, while after modification the thickness index increased to 226–232 μm. The calculated basis weight of the coated paper also increased and amounted to 162.9–163.9 g/m^2^ compared to the basis weight of 133.0 g/m^2^ for clean paper. As a result, the presence of a hydrophobic coating was confirmed by an increase in the thickness and basis weight of kraft paper after its hydrophobization by 23 wt.%. This indicates that the obtained hydrophobic paper is capable of exhibiting improved mechanical properties, which are discussed in the following sections.

#### 2.3.4. Resistance to Aggressive Environments and Temperatures

The durability of paper is one of the key parameters that determine its performance characteristics. However, the hydrophobic properties of the paper material can easily be adversely affected by harsh external factors, which can lead to the destruction of the hydrophobic coating [58]. Therefore, the obtained hydrophobic paper material (samples TFSQ_5%_, OFSQ_5%_, DFSQ_5%_) was studied for its resistance to environmental influences.

**Figure 4:**
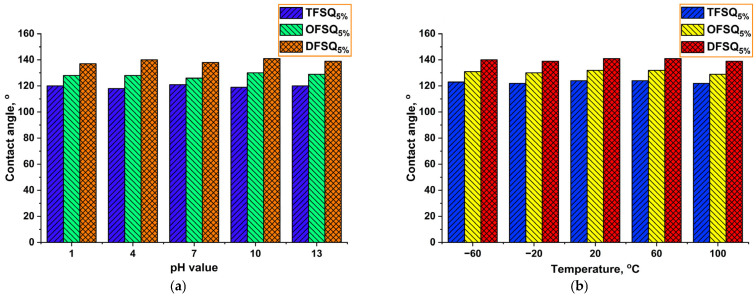
Dependence of contact angle on acidity of the medium (**a**) and temperature (**b**) of TFSQ_5%_, OFSQ_5%_, and DFSQ_5%_.

Stability tests were conducted under the action of aqueous solutions of different acidity to determine the pH stability of the coated kraft paper (Figure 4a). To achieve this, a paper sample was immersed in aqueous solutions with pH values from 1 to 13 in increments of 3 pH units for 1 h, after which the WCA was measured as an assessment of resistance to the pH environment.

The obtained results showed that when coated kraft paper was exposed to aqueous solutions of strong acids and alkalis, the values of the contact angle remained virtually unchanged and were close to the initial values. For example, for DFSQ_5%_, the WCA values after exposure in an aqueous solution with pH 1, 4, 7, 10 and 13 were 137°, 140°, 138°, 141° and 139°, respectively. The obtained WCA values are lower by about 2–3°, which is an adequate deviation. This indicates that the hydrophobic coating is firmly fixed in the volume of the cellulose matrix of the paper and testifies to the excellent stability of TFSQ_5%_, OFSQ_5%_ and DFSQ_5%_ against the action of aggressive environments.

During operation, hydrophobic kraft paper can also be exposed to various temperature effects; therefore, tests were carried out on the resistance of coated kraft paper to temperature changes. Paper samples TFSQ_5%_, OFSQ_5%_ and DFSQ_5%_ were subjected to temperature exposure in the range from −60 to 100 °C in 40 °C increments for 1 h. The test results are shown in Figure 4b. Under differing temperature exposure, a minimal change in the WCA was observed on the coated kraft paper, which was about 0.5°, compared to the initial values. For example, after exposure to temperatures at −60, −20, 20, 60 and 100 °C on a sample of DFSQ_5%_, the WCA values were 140°, 139°, 141°, 141° and 139°, respectively. Thus, the results of the tests indicate high thermal stability of the obtained highly hydrophobic kraft paper, and also indicate that the effects of temperature do not lead to the decomposition of both the polymer coating and the kraft paper itself.

#### 2.3.5. Moisture Resistance Analysis

**Figure 5:**
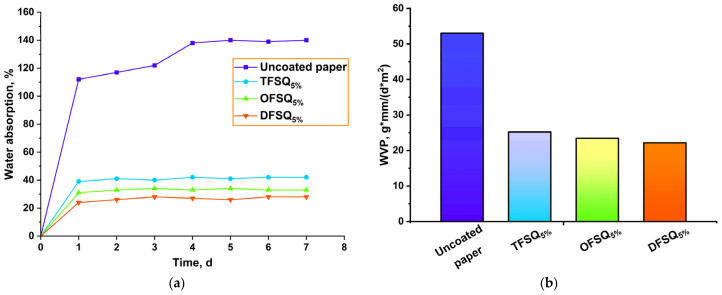
Time dependence of water absorption (**a**) and water vapor permeability (**b**) of uncoated kraft paper, TFSQ_5%_, OFSQ_5%_, and DFSQ_5%_.

To evaluate the water absorption capacity [59], the hydrophobic and uncoated kraft paper substrates were immersed in deionized water for 7 days, and the water absorption value was measured every day. The test results are shown in Figure 5a.

The obtained test results showed that the water absorption of all paper samples was obviously increased after immersion in water for one day, which was due to the interaction of free hydroxyl groups of cellulose with water, which led to swelling of the paper [60]. After four days, the water absorption of uncoated kraft paper was 138% and remained virtually unchanged during further measurements, indicating a state of equilibrium due to complete swelling of the paper. In turn, the coated kraft paper samples showed better water resistance due to the hydrophobic nature of the paper, as evidenced by the low water absorption coefficient, which was ~41%, ~33%, and ~27% for TFSQ_5%_, OFSQ_5%_ and DFSQ_5%_, respectively.

The water vapor permeability or vapor barrier property is one of the key indicators in assessing the functional properties of packaging materials. The optimal value of the WVP ensures the maintenance of a balanced water vapor regime inside the package, which helps to preserve the freshness of products, prevent the formation of condensation and minimize the loss of aromatic compounds and moisture. At the same time, the vapor barrier properties of the paper material must be characterized by a minimum value of the WVP coefficient for effective protection of the contents from excessive evaporation and penetration of moisture from the environment [61]. In this regard, the WVP of the obtained hydrophobic kraft paper was studied under conditions of relative humidity of 76%, the results of which are shown in Figure 5b. It is known that the mechanism of water vapor permeability through the porous structure of paper is realized through successive processes of sorption, diffusion and desorption of water vapor [62]. The obtained results showed that the WVP of the uncoated kraft paper was 53.03 g⋅mm/(d⋅m^2^), which is highly permeable to water vapor due to the porous structure of the paper, as shown in the SEM image (Figure 2). The WVP value of coated kraft paper samples was significantly reduced compared to uncoated paper by 53%, 56%, and 58% for TFSQ_5%_, OFSQ_5%_, and DFSQ_5%_, respectively, which is undoubtedly due to its hydrophobic nature. These observations are in good agreement with the results of SEM analysis, which showed that hydrophobic kraft paper has a smoother and more uniform surface morphology due to the filling and masking of pores with polymer macromolecules, which led to a noticeable decrease in vapor permeability. These results also agree well with the above-described water absorption test, in which hydrophobic kraft paper demonstrated excellent water resistance, which justifiably predetermined its high vapor barrier properties. Thus, the results of water absorption and vapor permeability studies showed that the obtained highly hydrophobic kraft paper has highly moisture-resistant properties, which opens up prospects for its use in humid conditions.

#### 2.3.6. Mechanical Strength

**Figure 6:**
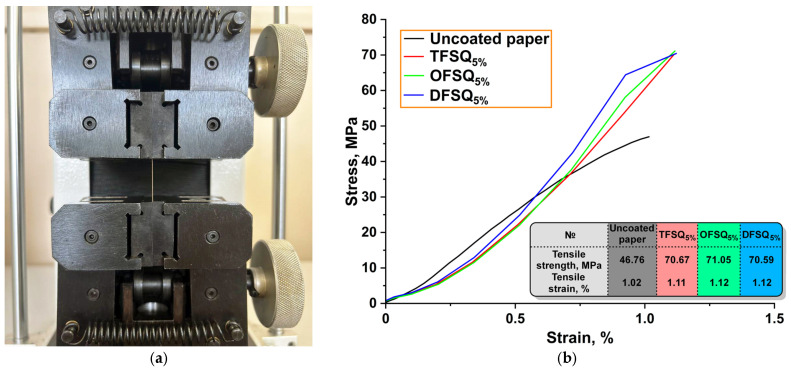
Photograph of hydrophobic kraft paper DFSQ_5%_ during stretching process (**a**) and stress–strain curve of uncoated kraft paper and TFSQ_5%_, OFSQ_5%_, and DFSQ_5%_ (**b**).

The mechanical strength of paper is a characteristic that determines its ability to withstand mechanical impacts during processing, storage and transportation, as well as to ensure the preservation of structural integrity when used as a packaging material [63]. Therefore, the mechanical properties of pure and hydrophobic paper were investigated to check their strength characteristics. The results of the stress–strain test are shown in Figure 6.

The obtained tensile results showed that after modification of kraft paper, its tensile strength increased by 24 MPa or 51.3%, reaching a value of 70.8 MPa (243.5 N), while for the uncoated kraft paper sample this indicator was 46.8 MPa (129.8 N). The increase in tensile strength after modification of kraft paper is due to the formation of a denser structure of cellulose fibers due to the introduced hydrophobic agent, which strengthened the cellulose structure. These results are in good agreement with the data presented in Table 3, which demonstrates an increase in the basis weight parameter of the paper material after modification. The improvement in the mechanical characteristics of the coated kraft paper is probably due to the condensation reaction, accompanied by the formation of covalent interfacial bonds between the polymer and the cellulose matrix. It is worth noting that the modification of the paper did not lead to a significant change in the relative tensile strain, the value of which was about 1.09%. Consequently, the obtained results of mechanical strength testing are important for the development of highly effective paper materials and open up prospects for the use of the obtained highly hydrophobic kraft paper in the development of new types of packaging materials with enhanced mechanical characteristics.

#### 2.3.7. Wear Resistance Analysis

Wear of packaging materials occurs mainly as a result of various physical damage that occurs during everyday use. Factors such as mechanical shocks, friction, and other external influences lead to a gradual deterioration in the structural integrity of packaging materials, which can negatively affect their functional and operational characteristics [64]. In this regard, the wear resistance of the obtained highly hydrophobic kraft paper was assessed by testing wettability after exposure to various mechanical loads, such as bending, finger-wipe and sandpaper abrasion.

**Figure 7:**
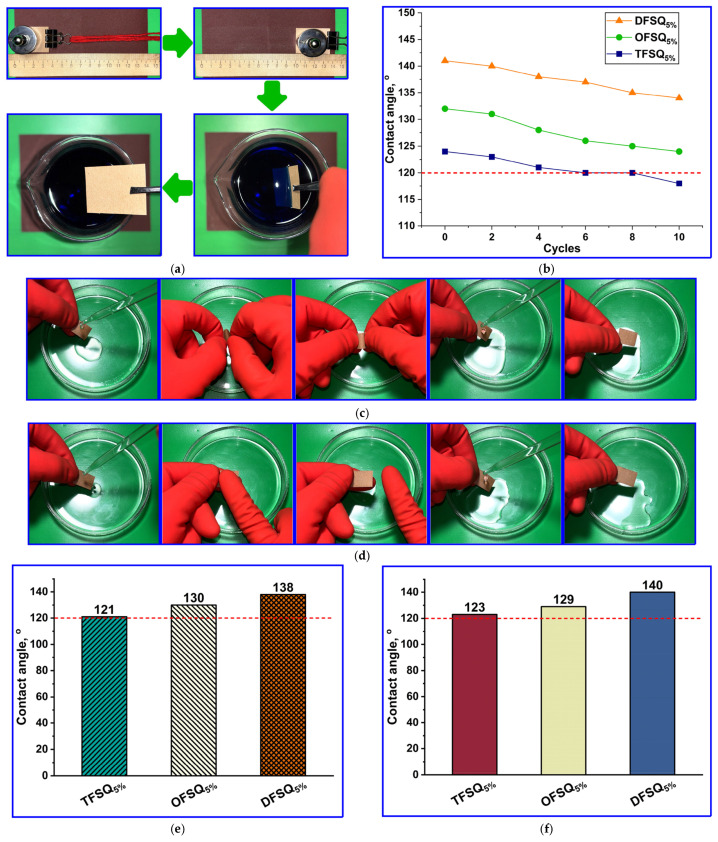
Abrasion (**a**), bending (**c**) and finger-wipe (**d**) tests of TFSQ_5%_, OFSQ_5%_ and DFSQ_5%_; relationship between WCA values and abrasion cycles (**b**) of TFSQ_5%_, OFSQ_5%_ and DFSQ_5%_; WCA values of TFSQ_5%_, OFSQ_5%_ and DFSQ_5%_ after 50 cycles of bending (**e**) and finger-wipe (**f**).

Figure 7a shows the abrasion test procedure. A hydrophobic kraft paper sample was placed on sandpaper under a 100 g weight and moved 10 cm along the ruler. Such a movement was defined as one abrasion cycle.

After 10 cycles of abrasive effects, the hydrophobic paper samples (TFSQ_5%_, OFSQ_5%_ and DFSQ_5%_) were immersed for 30 s in an aqueous solution of methylene blue dye used as a pollutant. After extraction from the solution, the kraft paper still demonstrated highly water-repellent properties, although it was partially damaged after rubbing with sandpaper. Figure 7b shows the results of WCA measurements of hydrophobic paper under different abrasion cycles. It can be seen that all paper samples retain highly hydrophobic properties after every two abrasion cycles. Even after 10 cycles of abrasive action, the WCA values remain above 120°, which indicates the high resistance of hydrophobic paper to abrasive action. Figure 7c,d show the bending test process and finger-wipe test process, respectively. During the experiment, hydrophobic kraft paper samples were subjected to back-and-forth bending at an angle of approximately 90° or finger-wiping in the longitudinal direction. A completed bend or wipe was defined as one cycle. After 50 cycles of bending and finger-wiping, the hydrophobic kraft paper samples (TFSQ_5%_, OFSQ_5%_ and DFSQ_5%_) were observed to retain their original shape and appearance without any visible damage. Moreover, despite the cyclic loads carried out, the kraft paper samples invariably retained their highly hydrophobic properties, as evidenced by the WCA values shown in Figure 7e,f. Thus, the obtained highly hydrophobic kraft paper demonstrated high wear resistance when exposed to various mechanical loads, such as bending, finger-wipe and sandpaper abrasion. The test results showed that modification with synthesized polymers not only provides hydrophobic properties of the paper material, but also enhances its mechanical characteristics. This opens up prospects for using this material in conditions where long-term operation and resistance to mechanical damage are required.

## 3. Materials and Methods

### 3.1. Materials

Commercially available initial reagents required to prepare the starting monomers for the synthesis of polyfluorinated silsesquioxane polymers included 2,2,3,3-tetrafluoropropan-1-ol (98%), 2,2,3,3,4,4,5,5-octafluoropentan-1-ol (98%), 2,2,3,3,4,4,5,5,6,6,7,7-dodecafluoroheptan-1-ol (98%), 3-bromoprop-1-ene (99%), hexachloroplatinic (IV) acid hexahydrate (39–41% Pt), triethoxysilane (95%), sodium hydroxide (NaOH, ≥98%, pellets (anhydrous)), and ethanol (95%), which were purchased from Sigma-Aldrich (Munich, Germany) and used as received. Propan-2-ol was distilled over anhydrous calcium sulfate immediately before use to prepare a 6% Speier catalyst solution. Tetrahydrofuran was dried over sodium benzophenone ketyl and distilled before use. Deionized water (resistivity ≥ 17.5 MΩ∙cm, Vodoley-M water purifier, Moscow, Russia) was used to prepare an aqueous solution of sodium hydroxide. Kraft paper (130 g/m^2^) was purchased at the nearest supermarket.

### 3.2. Synthesis

#### 3.2.1. General Technique of Synthesis of Polyfluorinated Silsesquioxane Polymers (FSQs)

Sodium hydroxide (0.5 M, 1.8 g, 0.1 mol) was added dropwise under stirring in an Ar atmosphere into a three-necked round-bottom flask (30 mL) containing 10 mL of an absolute ethanol solution of the corresponding triethoxy[3-(polyfluoroalkoxy)propyl]silane (FTSs) (0.01 mol). Next, the reaction mixture was vigorously stirred at 70 °C for 20 h. Then, the solvent was distilled off in vacuo. After that, the resulting colorless solid was washed with deionized water and dried in a vacuum oven at 30 °C to a constant weight for 24 h.

[3-(2,2,3,3-Tetrafluoropropoxy)propyl]silsesquioxane (TFSQ): The compound was obtained using 3.36 g triethoxy[3-(2,2,3,3-tetrafluoropropoxy)propyl]silane (TFTS). The yield of the target TFSQ was 2.16 g (91%). All characterization data, including FTIR and NMR spectra, are presented in the Supplementary Materials.

{3-[(2,2,3,3,4,4,5,5-Octafluoropentyl)oxy]propyl}silsesquioxane (OFSQ): The compound was obtained using 4.36 g (0.01 mol) of triethoxy{3-[(2,2,3,3,4,4,5,5-octafluoropentyl)oxy]propyl}silane (OFTS). The yield of the target OFSQ was 3.09 g (95%). All characterization data, including FTIR and NMR spectra, are presented in the Supplementary Materials.

{3-[(2,2,3,3,4,4,5,5,6,6,7,7-Dodecafluoroheptyl)oxy]propyl}silsesquioxane (DFSQ): The compound was obtained using 5.36 g (0.01 mol) of {3-[(2,2,3,3,4,4,5,5,6,6,7,7-dodecafluoroheptyl)oxy]propyl}(triethoxy)silane (DFTS). The yield of the target DFSQ was 3.95 g (93%). All characterization data, including FTIR and NMR spectra, are presented in the Supplementary Materials.

#### 3.2.2. Modification of Kraft Paper

Modification of kraft paper was carried out using a one-step method by immersing paper in ethanol solutions of FSQs. For the corresponding polysilsesquioxane TFSQ, OFSQ or DFSQ, a series of ethanol solutions with a concentration of 3 wt.%, 5 wt.% or 7 wt.% were prepared. Before immersion of the paper substrates, the solutions were stirred for 1 h. A medium-sized kraft paper sample was immersed in the resulting solution for 15 min, of which 5 min involved exposure to ultrasound (120 VA, 30%). The ultrasonic treatment was performed using a technological ultrasonic apparatus “Volna” with a frequency of mechanical vibrations of 22 ± 1.65 kHz and a maximum power consumption of 400 VA (Biysk, Russia). After this, it was removed and cleaned of excess ethanol, then dried at 60 °C for 2 h, then at 120 °C for 1 h. For simplicity, the further designations of coated kraft paper will be presented in the formats TFSQ_X%_, OFSQ_X%_, and DFSQ_X%_, where X corresponds to the polymer concentration. The original kraft paper was named uncoated kraft paper.

FTIR for TFSQ_3%_, TFSQ_5%_ and TFSQ_7%_ (solid, ν, cm^–1^): 3340 (O–H) 2922, 2851 (C–H ν_s_ and ν_as_), 1459, 1427 (C–H δ_CH_), 1204 (C–F), 1117 and 1050 (Si–O–Si), 1050 and 1030 (C–O), 825 (Si–O–C). FTIR for OFSQ_3%_, OFSQ_5%_ and OFSQ_7%_ (solid, ν, cm^–1^): 3342 (O–H) 2915, 2850 (C–H ν_s_ and ν_as_), 1458, 1423 (C–H δ_CH_), 1202 (C–F), 1119 and 1048 (Si–O–Si), 1048 and 1029 (C–O), 814 (Si–O–C). FTIR for DFSQ_3%_, DFSQ_5%_ and DFSQ_7%_ (solid, ν, cm^–1^): 3344 (O–H) 2921, 2855 (C–H ν_s_ and ν_as_), 1455, 1424 (C–H δCH), 1205 (C–F), 1120 and 1051 (Si–O–Si), 1051 and 1032 (C–O), 814 (Si–O–C).

### 3.3. Paper Performance Characteristic Testing

#### 3.3.1. Water Contact Angle Analysis

To investigate the hydrophobic properties of the surface of each paper type, the WCA was measured using a contact angle instrument (DSA25S, Kruss Ltd., Hamburg, Germany) at room temperature, and the drop volume of deionized water was 1 μL. The WCA values were obtained by performing measurements five times at several different random locations on the paper sample and are presented as the arithmetic mean value in degrees.

#### 3.3.2. Water Absorption

The water absorption of the paper was calculated based on the gravimetric method [59]. Each paper was weighed in a dry state and then immersed in deionized water. After immersion for a certain period of time, the paper was removed, cleaned of excess water with filter paper and weighed. The water absorption (WA) was calculated using Equation (1):

(1) $$WA\left(\%\right)=\frac{W_{s}-W_{0}}{W_{0}}\times 100\%,$$

where W_s_ is the weight of paper after absorbing the free water (g); W_0_ is the initial weight of the paper (g).

#### 3.3.3. Water Vapor Permeability

The water vapor permeability, expressed in g⋅mm/(d⋅m^2^), is the rate at which moisture passes through a paper sample. To determine the WVP value, a 10 mm diameter paper sample was sealed on the top of a bottle containing pre-dried anhydrous calcium chloride and weighted. Then, the flask was placed in a desiccator where conditions of 76% relative humidity, created by a saturated aqueous solution of sodium chloride, and 25 °C were maintained. After 24 h, the flask was weighed and the WVP value of the paper sample was calculated using Equation (2) [65,66]:

(2) $$WVP(g\cdot mm/d\cdot m^{2})=\frac{∆m\times h}{∆t\times S},$$

where Δm is the weight change of the flask (g); h is the thickness of paper (mm); Δt is the time change during the testing process (d); S is the area of the test section of the paper (m^2^).

#### 3.3.4. Thickness, Basis Weight and Coating Load of Kraft Paper

The thickness of each sample was measured using a digital micrometer (Schut, Groningen, The Netherlands) with a precision of 0.001 mm at 10 different random locations on the paper and presented as the arithmetic mean value in µm.

The basis weight (g/m^2^) was defined as the mass (g) per unit surface area of paper (m^2^), using a sample size of 24 × 12 mm. To determine the basis weight by the difference between the weights, the paper sample was weighed before and after modification. The basis weight (BW) was calculated using the following Equation (3):

(3) $$BW\;\left(g/m^{2}\right)=\frac{weight}{area}.$$

The coating load (CL) is the difference between the basis weight of the paper after modification and the basis weight of the paper before modification, which was calculated using Equation (4):

(4) $$CL\left(g/m^{2}\right)={BW}_{\mathrm{coated}\mathrm{paper}}-{BW}_{\mathrm{uncoated}\mathrm{paper}}.$$

The coating load in wt.% was calculated as the difference between the weight (g) of the paper after modification and before modification, and then the resulting difference was divided by the weight of the paper before modification, as shown in Equation (5).

(5) $$CL\left(wt.\%\right)=\frac{W_{\mathrm{coated}\mathrm{paper}}-W_{\mathrm{uncoated}\mathrm{paper}}}{W_{\mathrm{uncoated}\mathrm{paper}}}\times 100\%.$$

#### 3.3.5. Mechanical Property Measurements

Tensile strength testing of the paper was conducted on a universal testing machine (TochPribor, Ivanovo, Russia) with a 0.5 kN load cell. The paper was cut into rectangular strips with dimension of 15 × 25 mm. The tensile experiments were conducted at room temperature, and the strain rate was set at 30 mm/min. Three tested strips of each sample were used, and the data were obtained from the average value of three repetitions.

### 3.4. Fourier Transform Infrared (FTIR) Spectroscopy

Fourier transform infrared spectra were obtained on a Spectrum Two FTIR spectrometer (PerkinElmer, Shelton, CT, USA) using the attenuated total reflectance (ATR) attachment at room temperature. The scanned wave-number range was 4000–500 cm^−1^ with a spectral resolution of 4 cm^–1^ and 16 scans.

### 3.5. NMR Spectroscopy

Multinuclear magnetic resonance ^1^H (400.13 MHz), ^13^C (100.62 MHz), ^19^F (376.50 MHz), and ^29^Si (79.50 MHz) spectra were recorded on a Bruker DPX-400 spectrometer (Bruker, Bremen, Germany) using CDCl_3_, DMSO-*d*_6_ or (CD_3_)_2_CO as solvent at room temperature. ^1^H and ^13^C chemical shifts were referenced to residual solvent resonances as an internal standard, ^19^F chemical shifts relative to the external standard CCl_3_F, and ^29^Si chemical shifts relative to Me_4_Si as an external standard.

### 3.6. Gel Permeation Chromatography (GPC)

The GPC measurements were carried out using a Shimadzu LC-20 Prominence system (Shimadzu Corporation, Kyoto, Japan) equipped with a Shimadzu RID-20A differential refractive index detector and an Agilent PolyPore 7.5 × 300 mm column (PL1113-6500). A weighed portion of the sample was preliminarily dissolved in tetrahydrofuran (THF) at room temperature with constant stirring for 24 h. The solution concentration was about 10 mg/mL. GPC analyses of all samples in THF solutions were performed at a flow rate of 1 mL/min at 50 °C. The samples were calibrated using polystyrene standards Polystyrene High EasiVials (PL2010-0201) with molecular weights ranging from 162 to 6,570,000 g/mol.

### 3.7. Thermogravimetric Analysis (TGA)

Thermogravimetric analysis was carried out on the TGA i 1000 Thermogravimetric Analyzer (Instrument Specialist incorporated, Twin Lakes, WI, USA). The experiment was conducted by heating a 10 mg sample at a rate of 10 °C per minute in the temperature range from 20 to 800 °C in an air flow.

### 3.8. Scanning Electron Micrograph (SEM) Analysis

Scanning electron microscopy images of the modified glass and paper were acquired using a TM3000 instrument from Hitachi (Tokyo, Japan).

## 4. Conclusions

Kraft paper was successfully modified by a one-step dip coating method using polyfluorinated polysilsesquioxanes synthesized by us as hydrophobic agents, and its hydrophobic properties and performance characteristics were studied. Functional silsesquioxane polymers with linear polyfluorinated side-chain substituents were easily obtained in high yield by a base-catalyzed hydrolytic polycondensation reaction of alkoxysilanes and characterized by a set of physicochemical analysis methods. It has been shown that the hydrophobic properties of kraft paper depend on the length of the fluoroalkyl chain of the substituent at the silicon atom and increase with an increase in the number of CF_2_ units in the polymer’s chemical structure. It has been established that a 5% polymer concentration in the solution for modifying kraft paper is optimal and gives it highly hydrophobic properties, while the contact angle values are maximum and reach 141°. According to FTIR spectroscopy data, covalent bonds are formed between the polymer and the cellulose matrix of the paper, ensuring the formation of a durable and stable coating. The polymer forms a favorable morphology on the paper surface, providing it with highly hydrophobic properties, which is confirmed by scanning electron microscopy. The thickness and basis weight of the paper after its modification increased by 23%. It is shown that the modified kraft paper has high resistance to chemically aggressive environments and temperature. The water absorption and water vapor permeability coefficients of the hydrophobized kraft paper decreased by more than 95% and 53%, respectively. After modification, kraft paper has a high resistance to mechanical wear, and, as a result, it retains its shape, appearance, and original highly hydrophobic properties after testing. The mechanical strength of modified kraft paper, compared to untreated paper, increased by 51%. The resulting highly hydrophobic kraft paper has great potential for use in the production of packaging materials with enhanced mechanical characteristics, long service life, and high resistance to the effects of liquids of various chemical natures, humid conditions, environmental impacts and physical damage, making them suitable for a wide range of multifunctional applications in different industries. The developed kraft paper can be used as a moisture-resistant packaging material for electronics, building materials, and industrial goods; as protective packaging for household chemicals and fertilizers; and as functional paper for technical purposes (e.g., labels, protective layers, etc.).

## Figures and Tables

**Table 1:** Average molecular weights and polydispersity indices of FSQs.

FSQ	M_n_, Da	M_w_, Da	Đ
TFSQ	12,200	16,800	1.38
OFSQ	14,700	20,900	1.42
DFSQ	12,400	17,300	1.40

**Table 2:** Water contact angle values of TFSQ-, OFSQ-, and DFSQ-coated kraft paper at different concentrations.

Sample	Concentration (X), wt.%		
	3	5	7
TFSQ_X%_	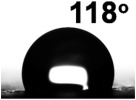	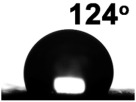	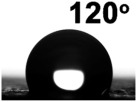
OFSQ_X%_	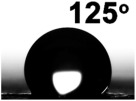	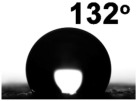	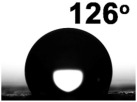
DFSQ_X%_	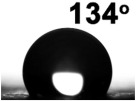	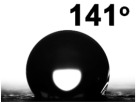	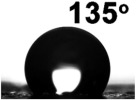

**Table 3:** Thickness, basis weight, and coating load of TFSQ_5%_, OFSQ_5%_, DFSQ_5%_, and uncoated kraft paper.

Sample	Material Thickness, μm	Basis Weight, g/m^2^	Coating Load, g/m^2^	Coating (Load by wt.%)
Uncoated kraft paper	185	133.0	0	0
TFSQ_5%_	226	162.9	30.3	22.9
OFSQ_5%_	230	163.5	30.5	23.0
DFSQ_5%_	232	163.9	30.9	23.2

## Data Availability

The original contributions presented in this study are included in the article and Supplementary Materials.

## Supplementary Materials

The supporting information can be downloaded at https://www.mdpi.com/article/10.3390/ijms262311719/s1.
