# Supplementary material for: Fluoro-Functionalized Silsesquioxane Polymer-Based High Hydrophobic Coatings for Enhancing Properties of Kraft Paper
Source: Int J Mol Sci. 2025 Dec 3;26(23):11719. doi: 10.3390/ijms262311719 (PMC12692189; doi:10.3390/ijms262311719)
Supplement: Supplementary file 1 [file ijms-26-11719-s001.zip › ijms-3979726-supplementary.pdf]

Supplementary materials

Table of Contents:

Figure S1. FTIR spectra of compounds **TFAE, OFAE, DFAE** S3

Figure S2. ^{1}H NMR spectrum of compound **TFAE** in $CDCl_3$ S3

Figure S3. ^{13}C NMR spectrum of compound **TFAE** in $CDCl_3$ S4

Figure S4. ^{19}F NMR spectrum of compound **TFAE** in $CDCl_3$ S4

Figure S5. ^{1}H NMR spectrum of compound **OFAE** in $CDCl_3$ S5

Figure S6. ^{13}C NMR spectrum of compound **OFAE** in $CDCl_3$ S5

Figure S7. ^{19}F NMR spectrum of compound **OFAE** in $CDCl_3$ S6

Figure S8. ^{1}H NMR spectrum of compound **DFAE** in $CDCl_3$ S6

Figure S9. ^{13}C NMR spectrum of compound **DFAE** in $CDCl_3$ S7

Figure S10. ^{19}F NMR spectrum of compound **DFAE** in $CDCl_3$ S7

Figure S11. FTIR spectra of compounds **TFTS, OFTS, DFTS** S8

Figure S12. ^{1}H NMR spectrum of compound **TFTS** in $CDCl_3$ S8

Figure S13. ^{13}C NMR spectrum of compound **TFTS** in $CDCl_3$ S9

Figure S14. ^{19}F NMR spectrum of compound **TFTS** in $CDCl_3$ S9

Figure S15. ^{29}Si NMR spectrum of compound **TFTS** in $CDCl_3$ S10

Figure S16. ^{1}H NMR spectrum of compound **OFTS** in $CDCl_3$ S10

Figure S17. ^{13}C NMR spectrum of compound **OFTS** in $CDCl_3$ S11

Figure S18. ^{19}F NMR spectrum of compound **OFTS** in $CDCl_3$ S11

Figure S19. ^{29}Si NMR spectrum of compound **OFTS** in $CDCl_3$ S12

Figure S20. ^{1}H NMR spectrum of compound **DFTS** in $CDCl_3$ S12

Figure S21. ^{13}C NMR spectrum of compound **DFTS** in $CDCl_3$ S13

Figure S22. ^{19}F NMR spectrum of compound **DFTS** in $CDCl_3$ S13

Figure S23. ^{29}Si NMR spectrum of compound **DFTS** in $CDCl_3$ S14

Characterization Data of **TFSQ** S14

Characterization Data of **OFSQ** S15

Characterization Data of **DFSQ** S15

Figure S24. FTIR spectra of compounds **TFSQ, OFSQ, DFSQ** S16

Figure S25. ^{1}H NMR spectrum of compound **TFSQ** in DMSO-d_6 S16

Figure S26. ^{13}C NMR spectrum of compound **TFSQ** in DMSO-d_6 S17

Figure S27. ^{19}F NMR spectrum of compound **TFSQ** in DMSO-d_6 S17

Figure S28. ^{29}Si NMR spectrum of compound **TFSQ** in DMSO-d_6 S18

Figure S29. ^{1}H NMR spectrum of compound **OFSQ** in DMSO-d_6 S18

Figure S30. ^{13}C NMR spectrum of compound **OFSQ** in DMSO-d_6 S19

Figure S31. ^{19}F NMR spectrum of compound **OFSQ** in DMSO-d_6 S19

Figure S32. ^{29}Si NMR spectrum of compound **OFSQ** in DMSO-d_6 S20

Figure S33. ^{1}H NMR spectrum of compound **DFSQ** in $(CD_3)_2CO$ S20

Figure S34. ^{13}C NMR spectrum of compound **DFSQ** in $(CD_3)_2CO$ S21

Figure S35. ^{19}F NMR spectrum of compound **DFSQ** in $(CD_3)_2CO$ S21

Figure S36. ^{29}Si NMR spectrum of compound **DFSQ** in $(CD_3)_2CO$ S22

Figure S37. GPC curves of **TFSQ**, **OFSQ**, and **DFSQ**. S22

Figure S38. TGA curves of **TFSQ**, **OFSQ**, and **DFSQ**. S23

Figure S39. EDS mapping of Si and F elements on the surface of **TFSQ$_{5\%}$** and **OFSQ$_{5\%}$**. S23

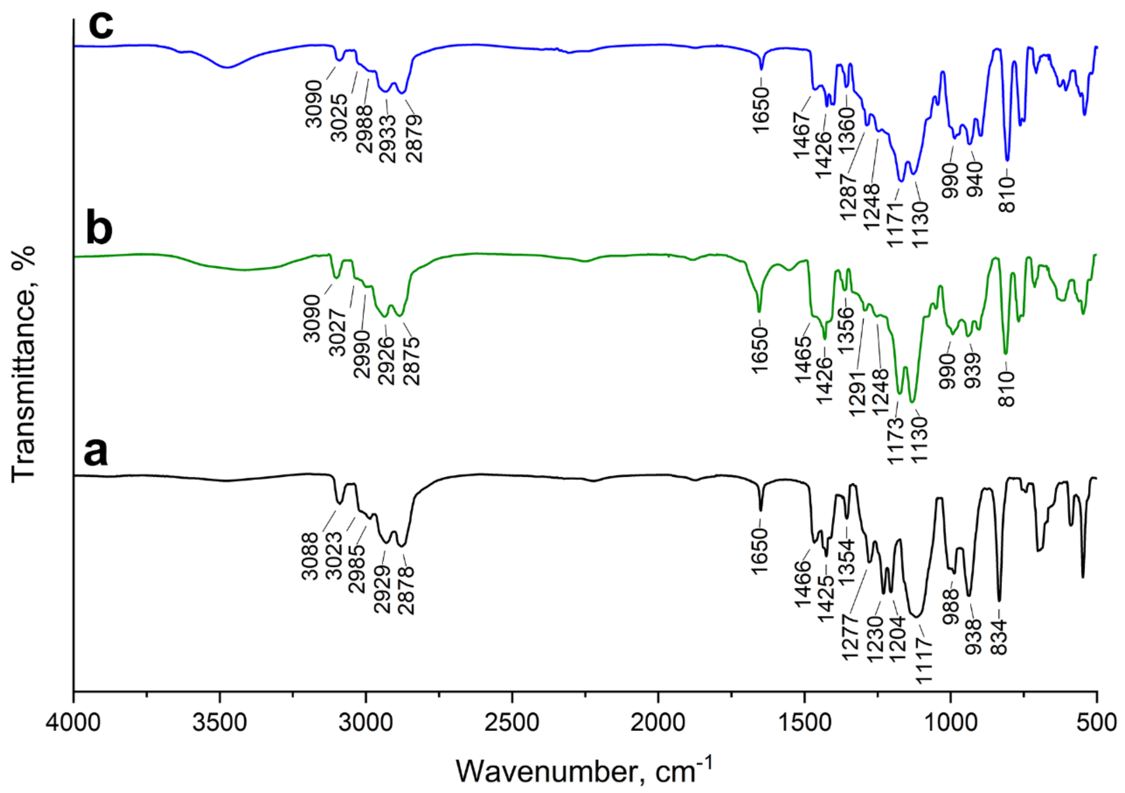

Figure S1. FTIR spectra of allyl 2,2,3,3-tetrafluoropropyl ether **TFAE** (a), allyl 2,2,3,3,4,4,5,5-octafluoropentyl ether **OFAE** (b), and allyl 2,2,3,3,4,4,5,5,6,6,7,7-dodecafluoroheptyl ether **DFAE** (c).

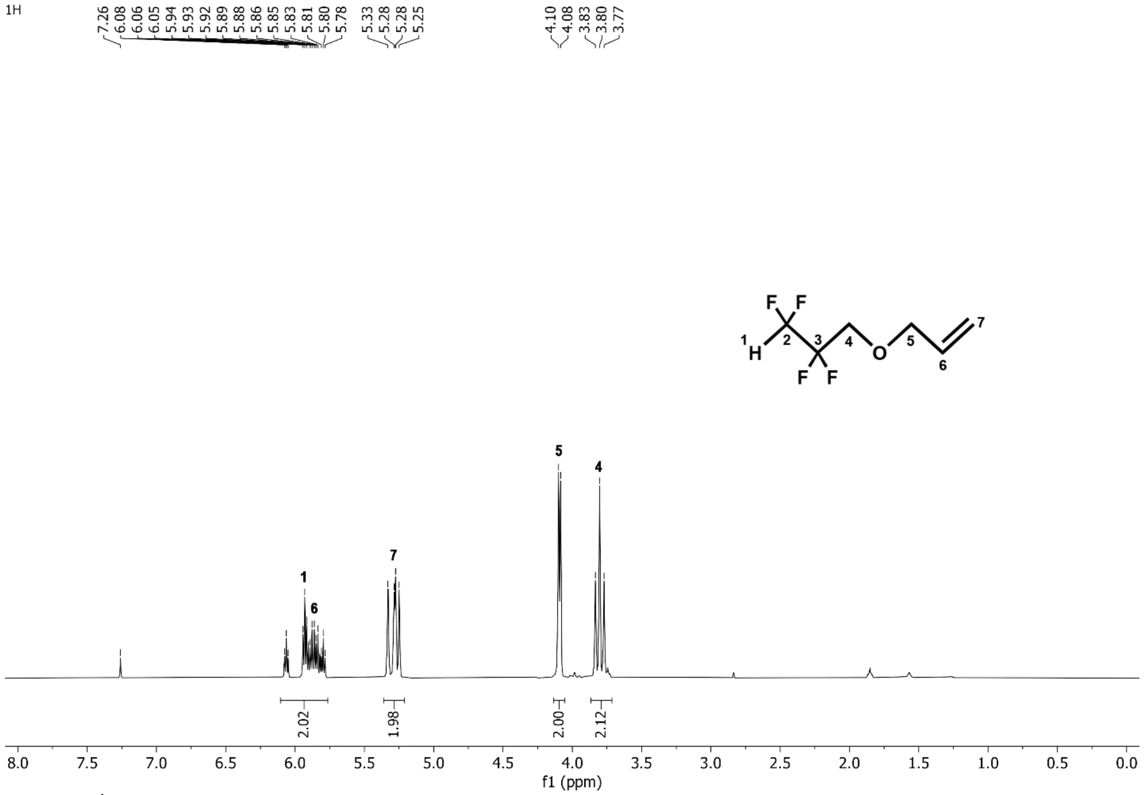

Figure S2. ^{1}H NMR spectrum of allyl 2,2,3,3-tetrafluoropropyl ether **TFAE** in $CDCl_3$.

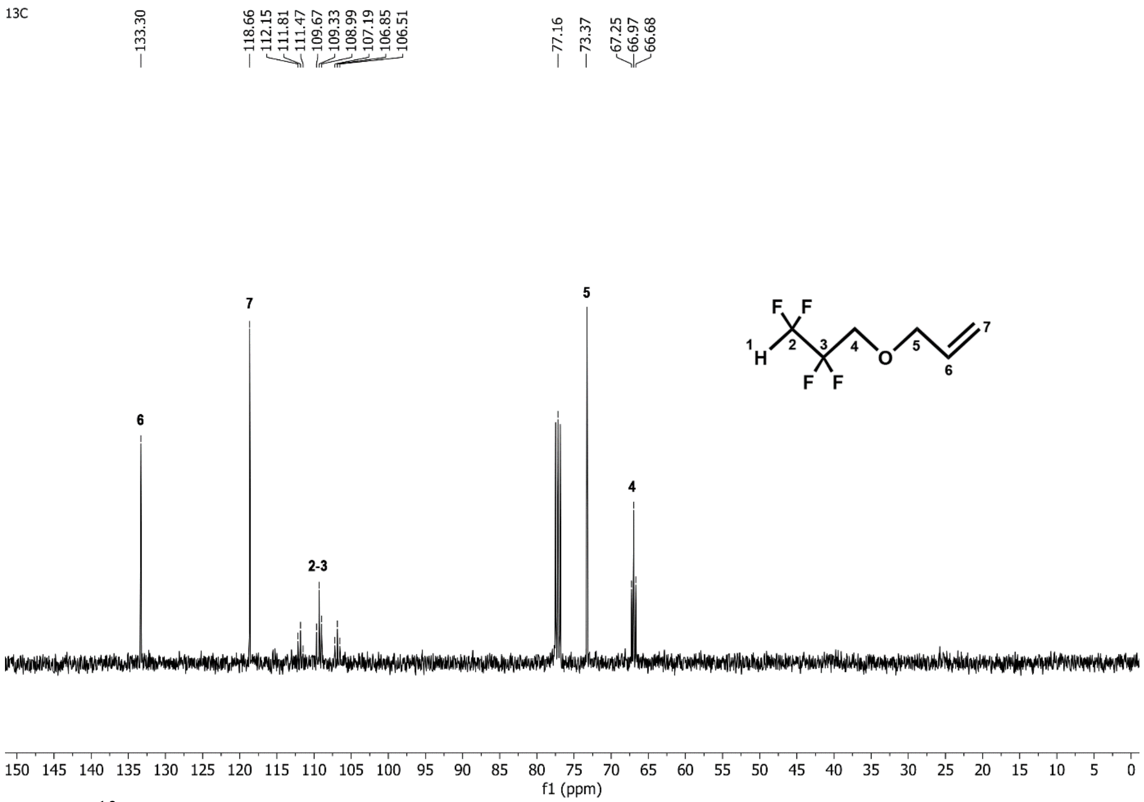

Figure S3. ^{13}C NMR spectrum of allyl 2,2,3,3-tetrafluoropropyl ether **TFAE** in $CDCl_3$.

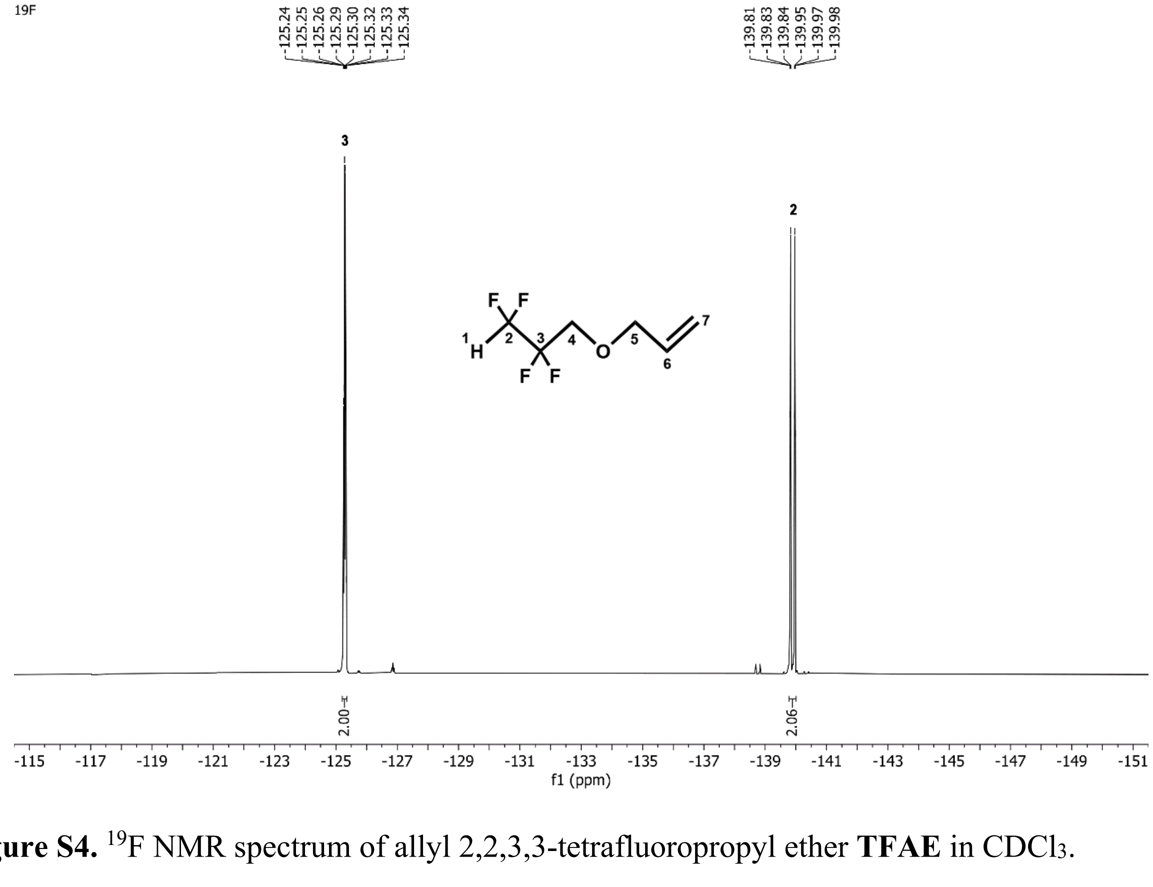

Figure S4. ^{19}F NMR spectrum of allyl 2,2,3,3-tetrafluoropropyl ether **TFAE** in $CDCl_3$.

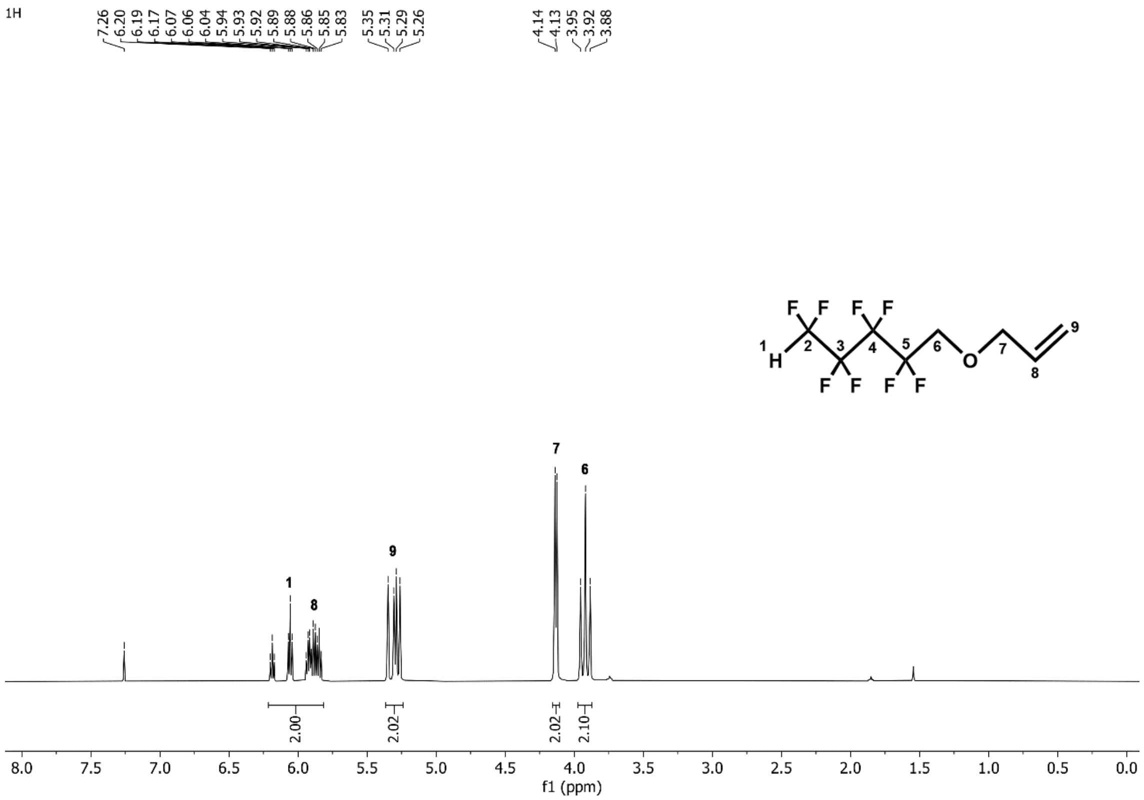

Figure S5. ^{1}H NMR spectrum of allyl 2,2,3,3,4,4,5,5-octafluoropentyl ether **OFAE** in $CDCl_3$.

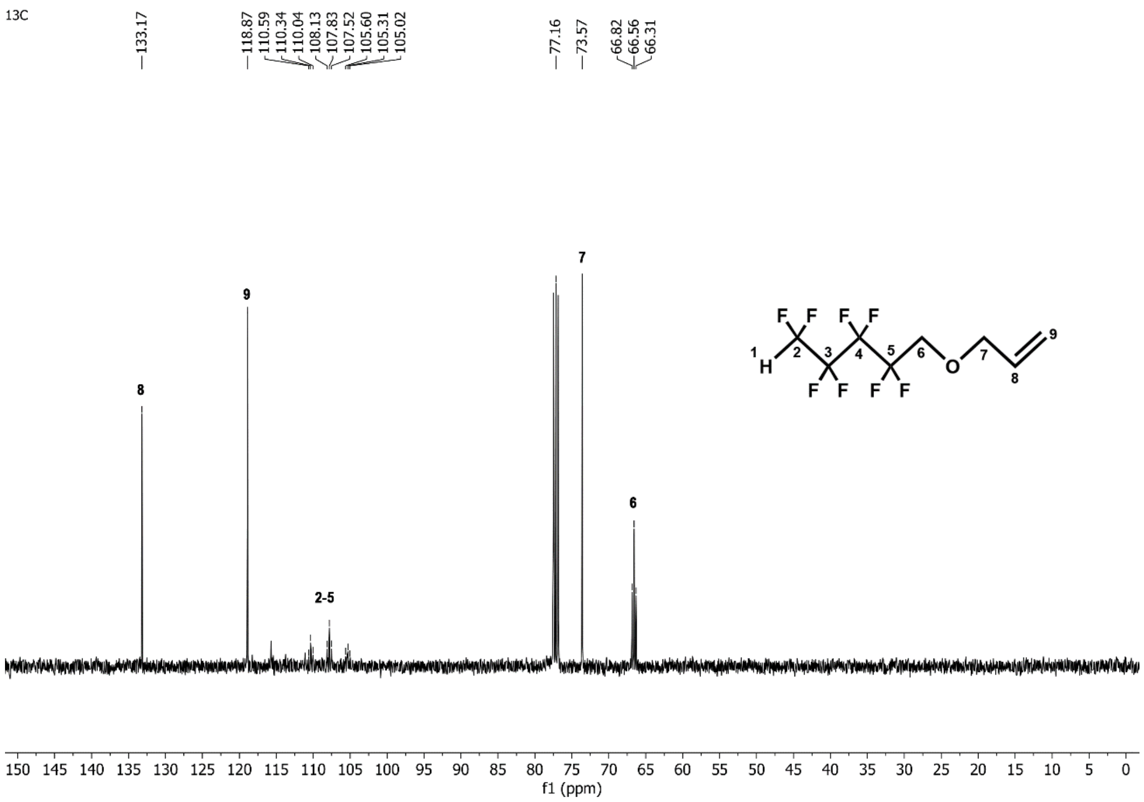

Figure S6. ^{13}C NMR spectrum of allyl 2,2,3,3,4,4,5,5-octafluoropentyl ether **OFAE** in $CDCl_3$.

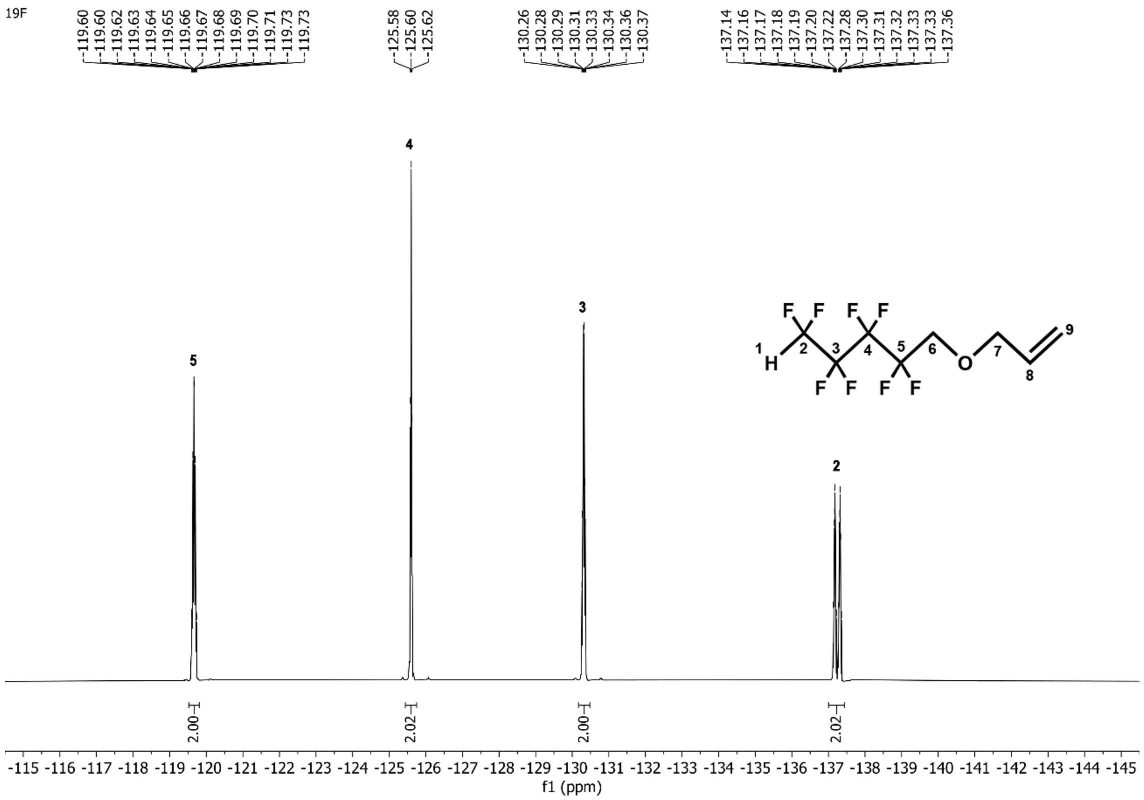

Figure S7. ^{19}F NMR spectrum of allyl 2,2,3,3,4,4,5,5-octafluoropentyl ether **OFAE** in $CDCl_3$.

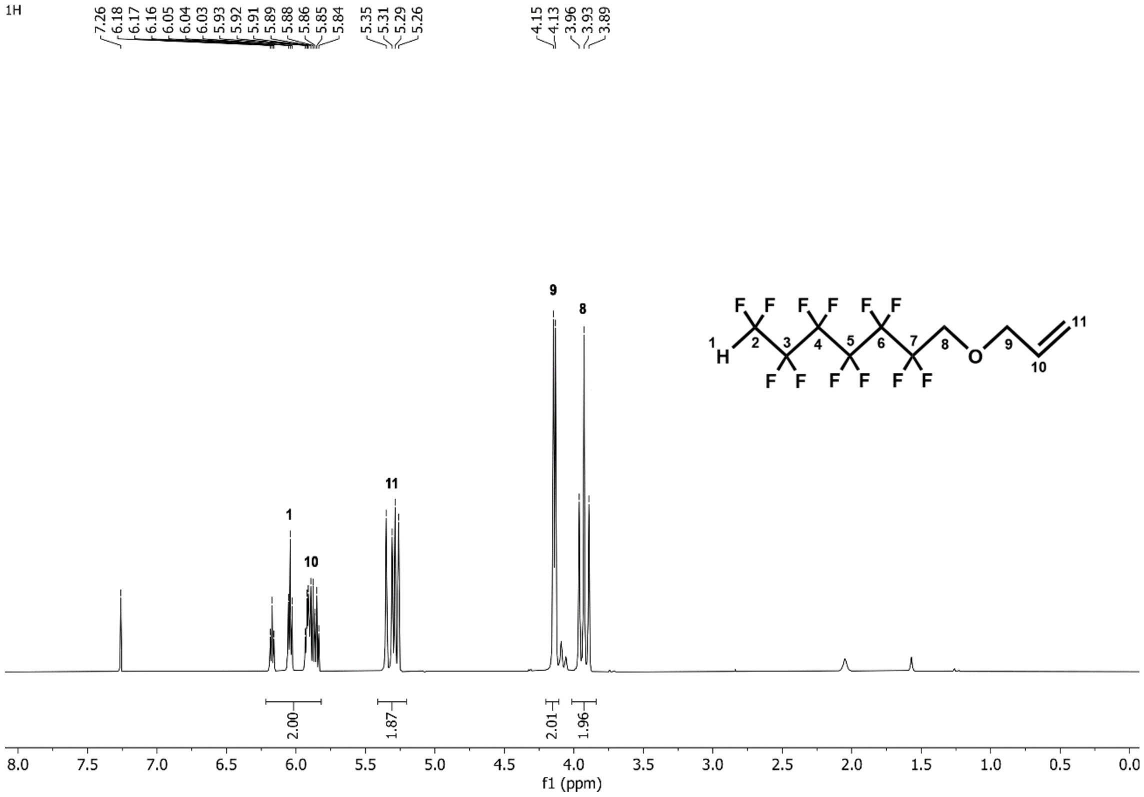

Figure S8. ^{1}H NMR spectrum of allyl 2,2,3,3,4,4,5,5,6,6,7,7-dodecafluorohepyl ether **DFAE** in $CDCl_3$.

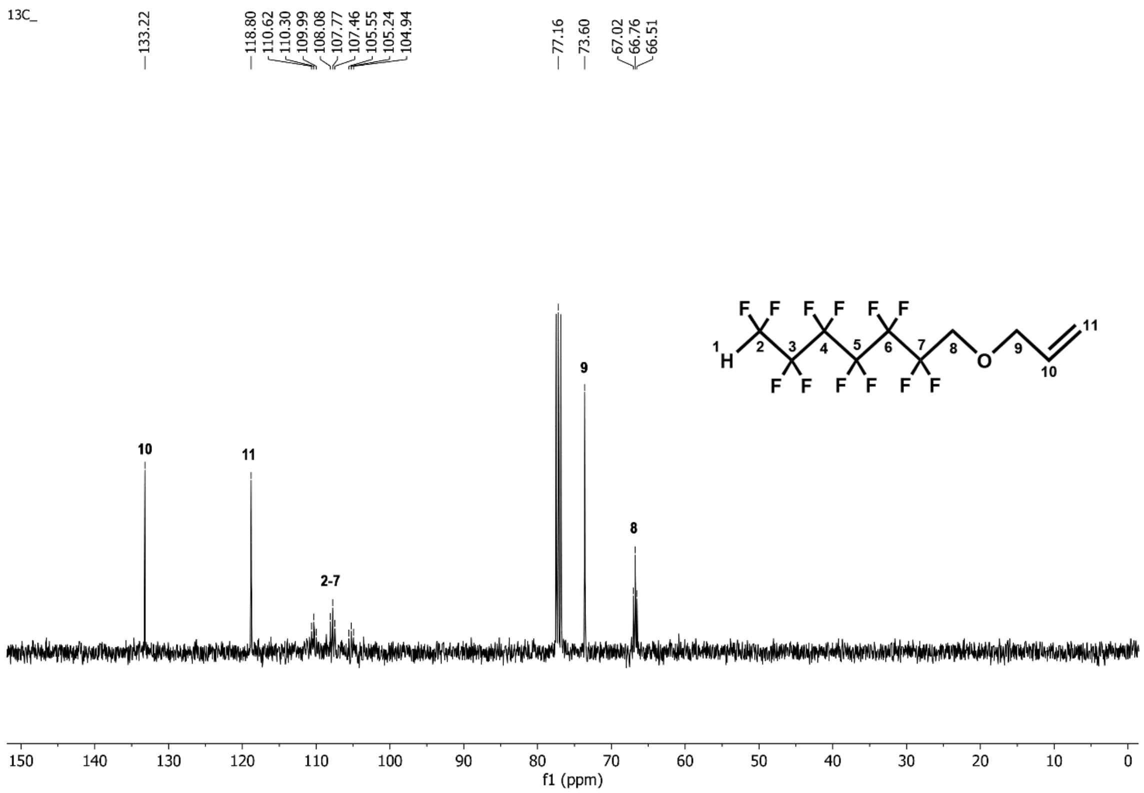

Figure S9. ^{13}C NMR spectrum of allyl 2,2,3,3,4,4,5,5,6,6,7,7-dodecafluorohepyl ether **DFAE** in $CDCl_3$.

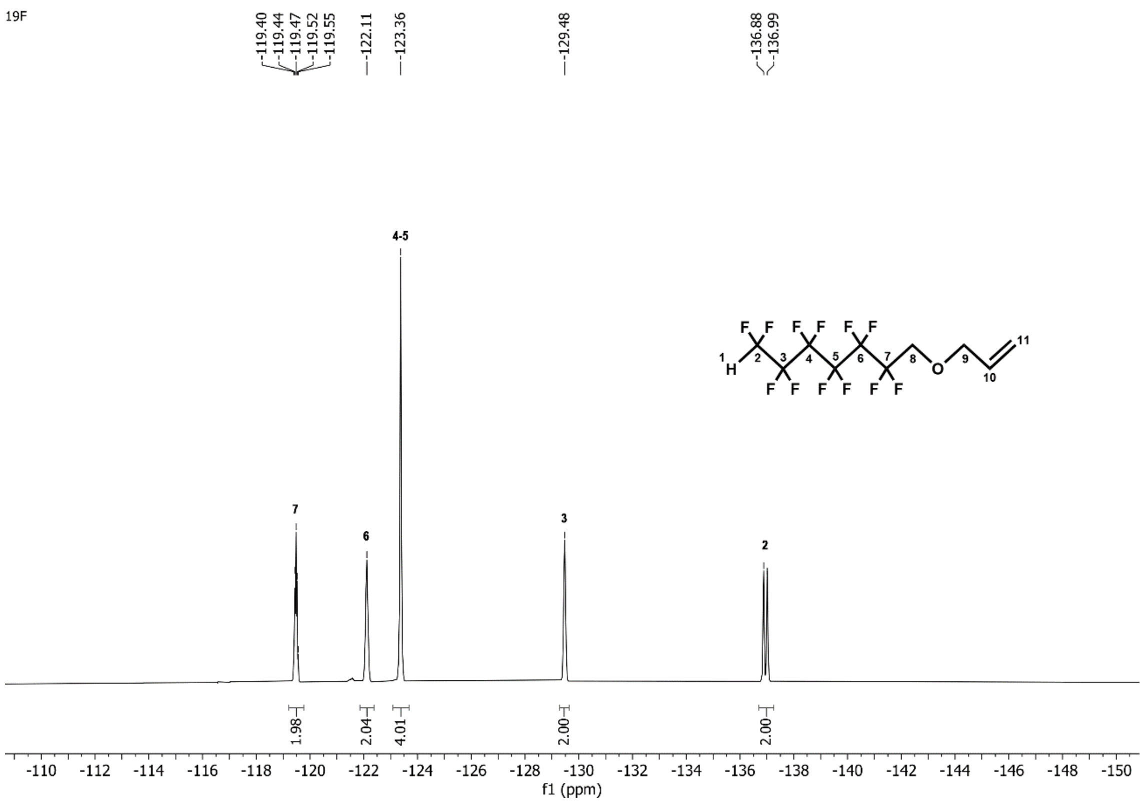

Figure S10. ^{19}F NMR spectrum of allyl 2,2,3,3,4,4,5,5,6,6,7,7-dodecafluorohepyl ether **DFAE** in $CDCl_3$.

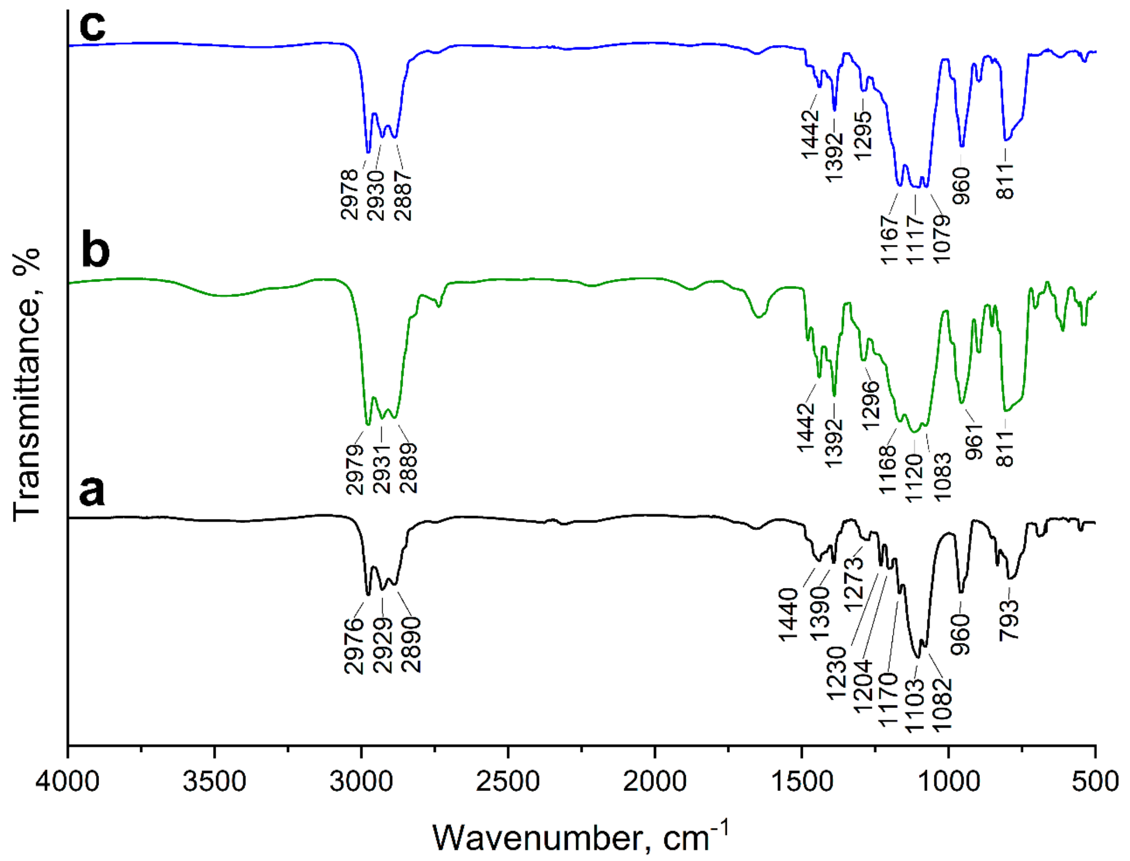

Figure S11. FTIR spectra of triethoxy[3-(2,2,3,3-tetrafluoropropoxy)propyl]silane **TFTS** (a), triethoxy{3-[(2,2,3,3,4,4,5,5-octafluoropentyl)oxy]propyl}silane **OFTS** (b), and {3-[(2,2,3,3,4,4,5,5,6,6,7,7-dodecafluoroheptyl)oxy]propyl}(triethoxy)silane **DFTS** (c).

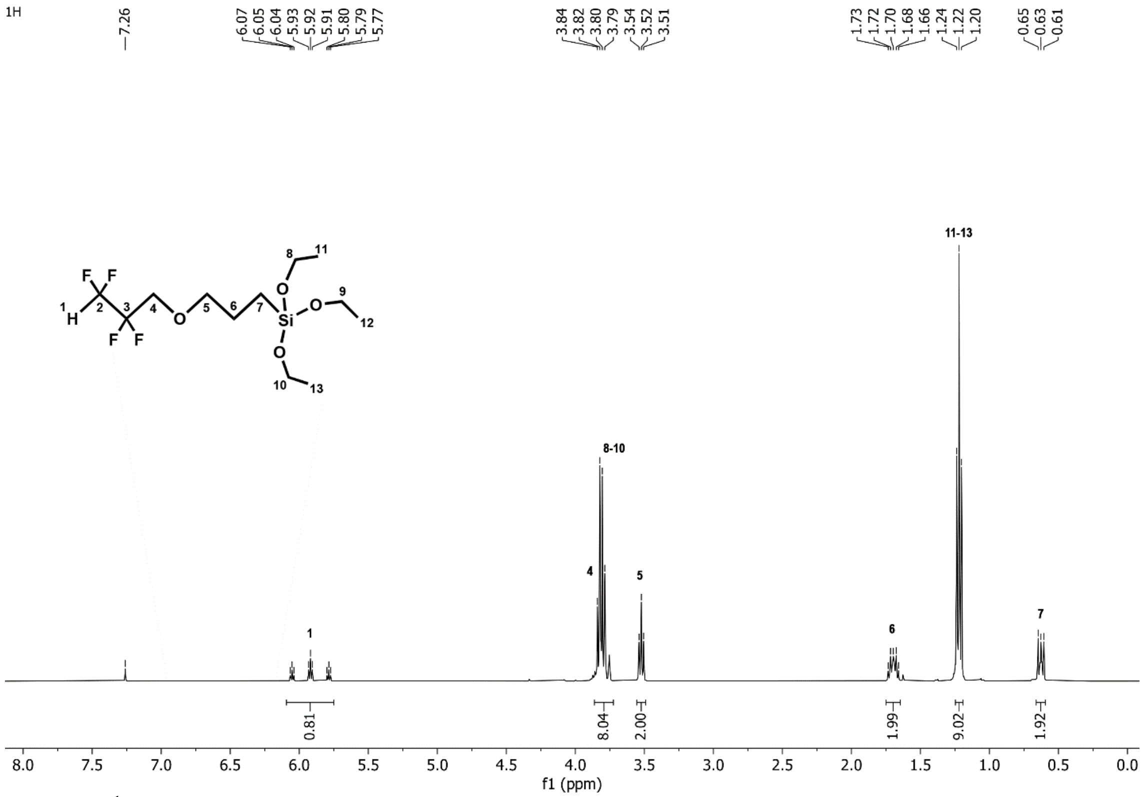

Figure S12. ^{1}H NMR spectrum of triethoxy[3-(2,2,3,3-tetrafluoropropoxy)propyl]silane **TFTS** in $CDCl_3$.

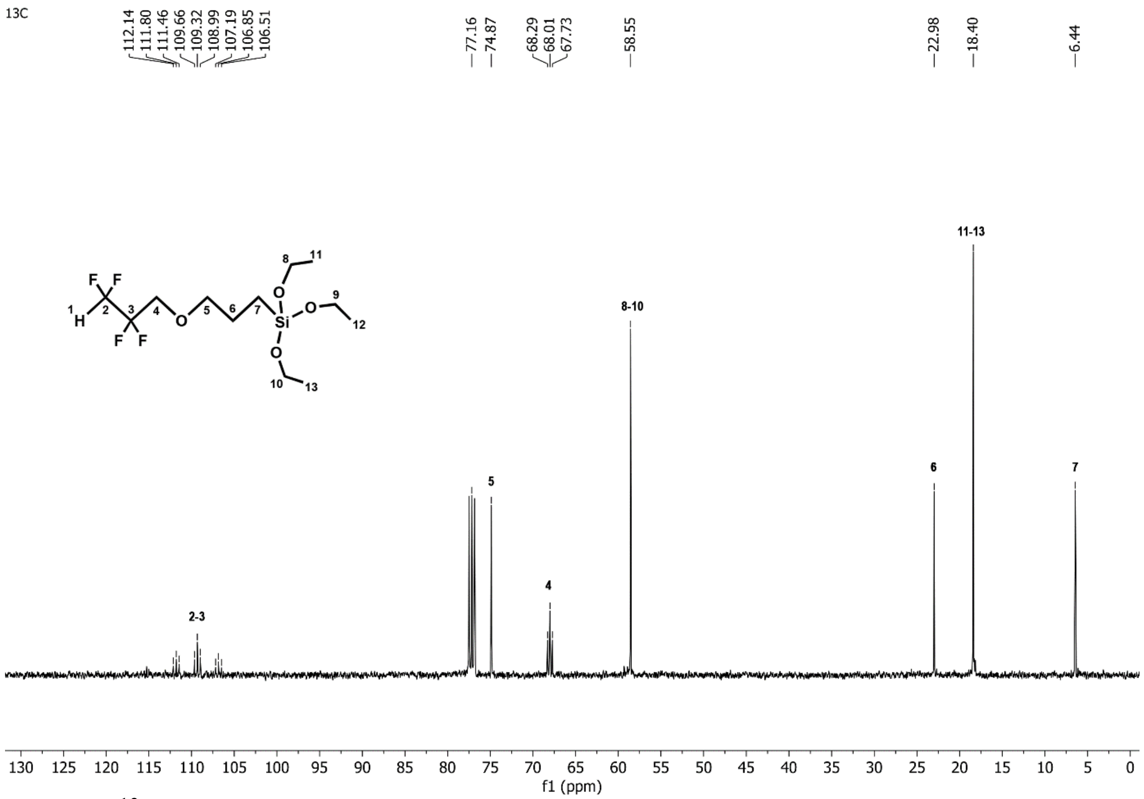

Figure S13. ^{13}C NMR spectrum of triethoxy[3-(2,2,3,3-tetrafluoropropoxy)propyl]silane **TFTS** in $CDCl_3$.

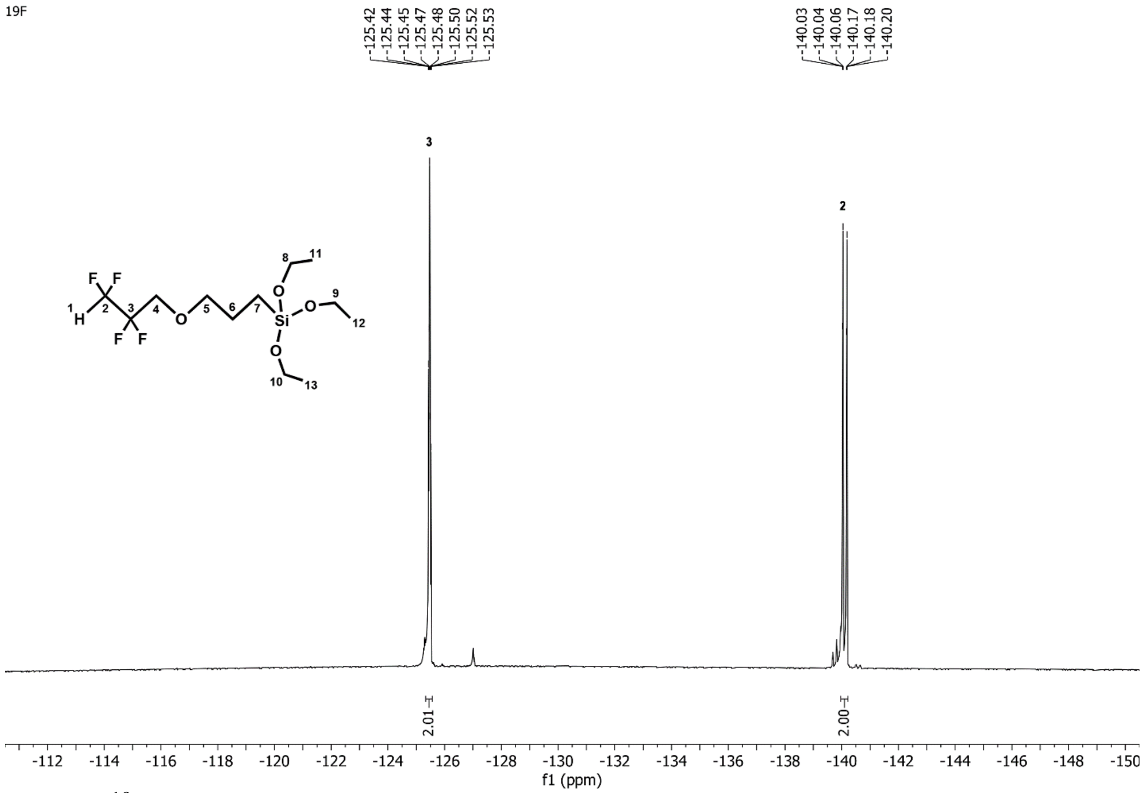

Figure S14. ^{19}F NMR spectrum of triethoxy[3-(2,2,3,3-tetrafluoropropoxy)propyl]silane **TFTS** in $CDCl_3$.

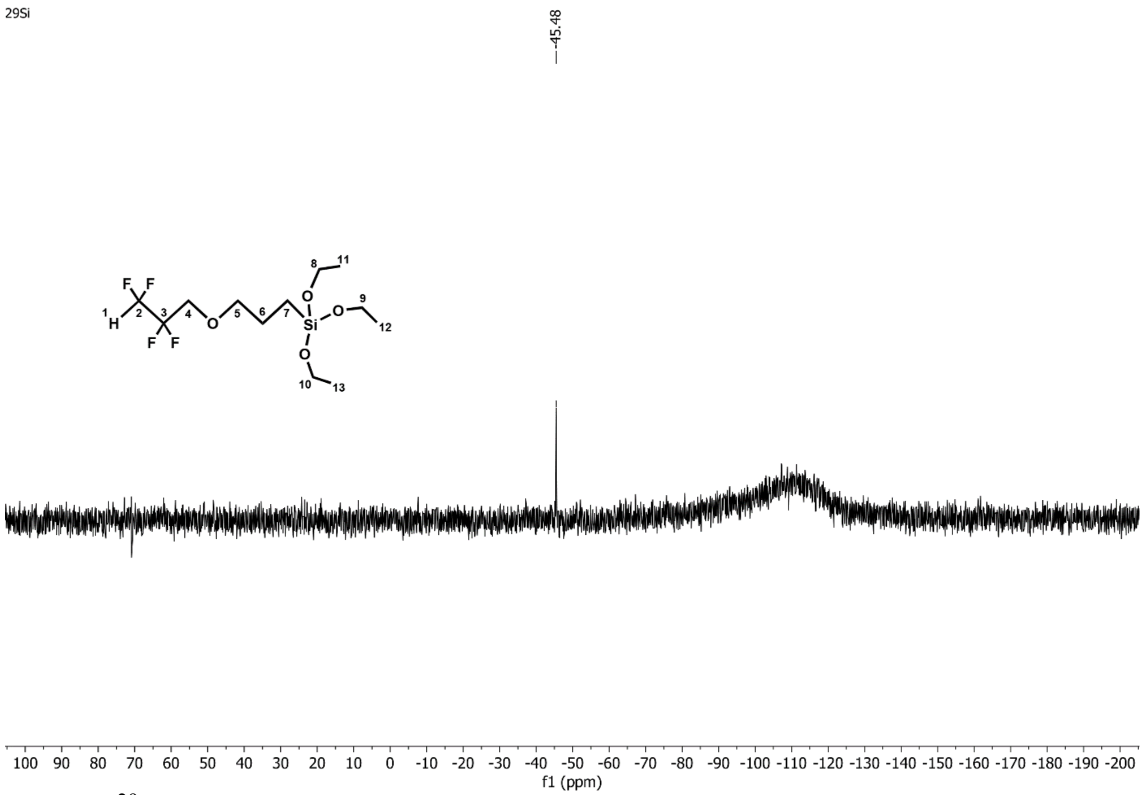

Figure S15. ^{29}Si NMR spectrum of triethoxy[3-(2,2,3,3-tetrafluoropropoxy)propyl]silane **TFTS** in $CDCl_3$.

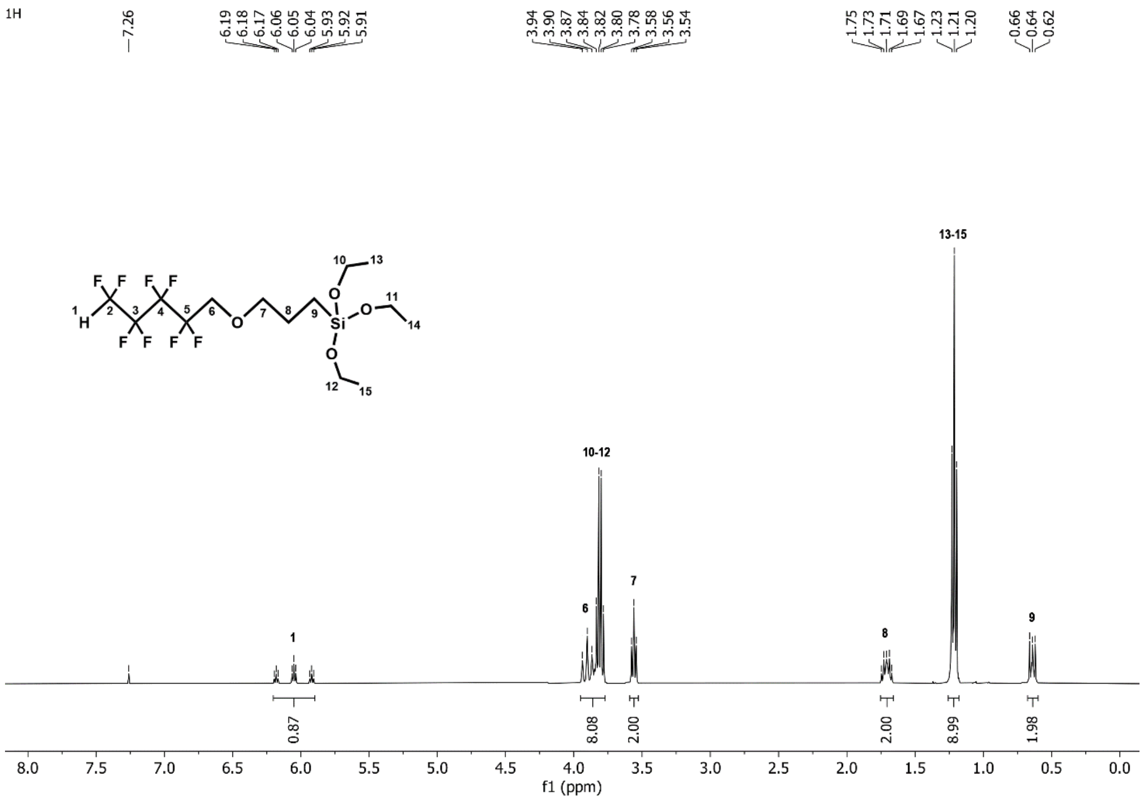

Figure S16. ^{1}H NMR spectrum of triethoxy{3-[(2,2,3,3,4,4,5,5-octafluoropentyl)oxy]propyl}silane **OFTS** in $CDCl_3$.

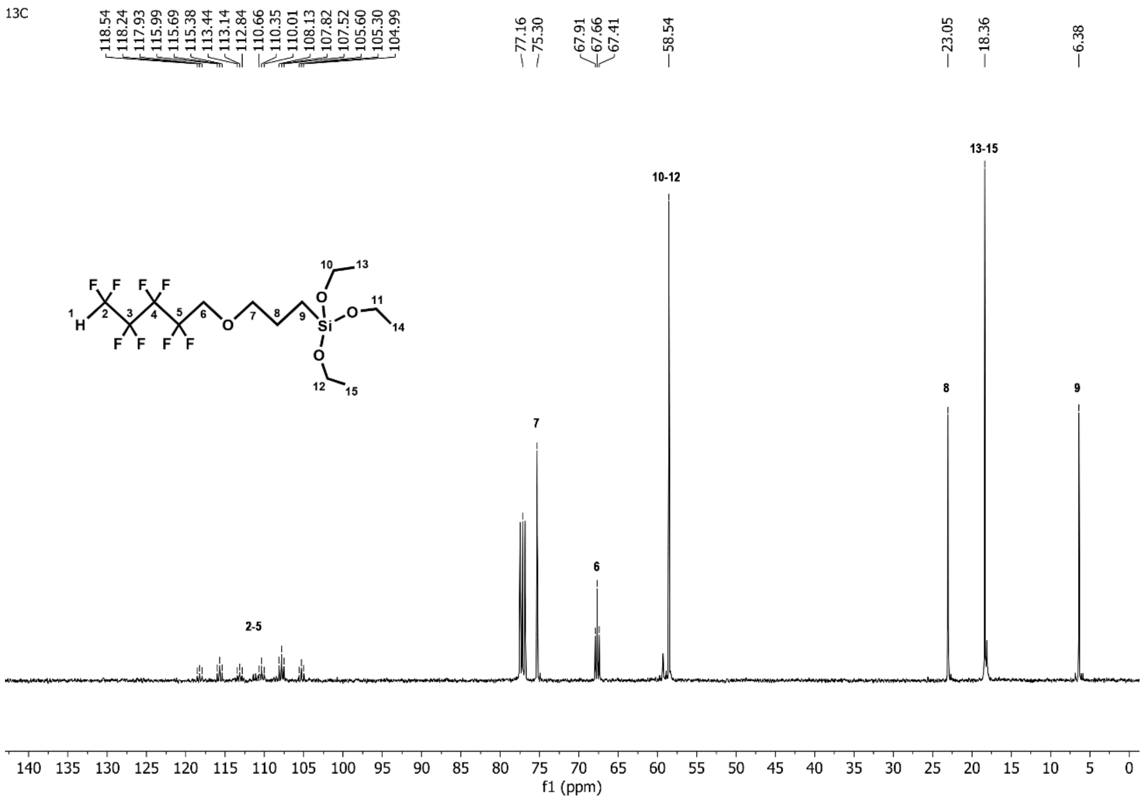

Figure S17. ^{13}C NMR spectrum of triethoxy{3-[(2,2,3,3,4,4,5,5-octafluoropentyl)oxy]propyl}silane **OFTS** in $CDCl_3$.

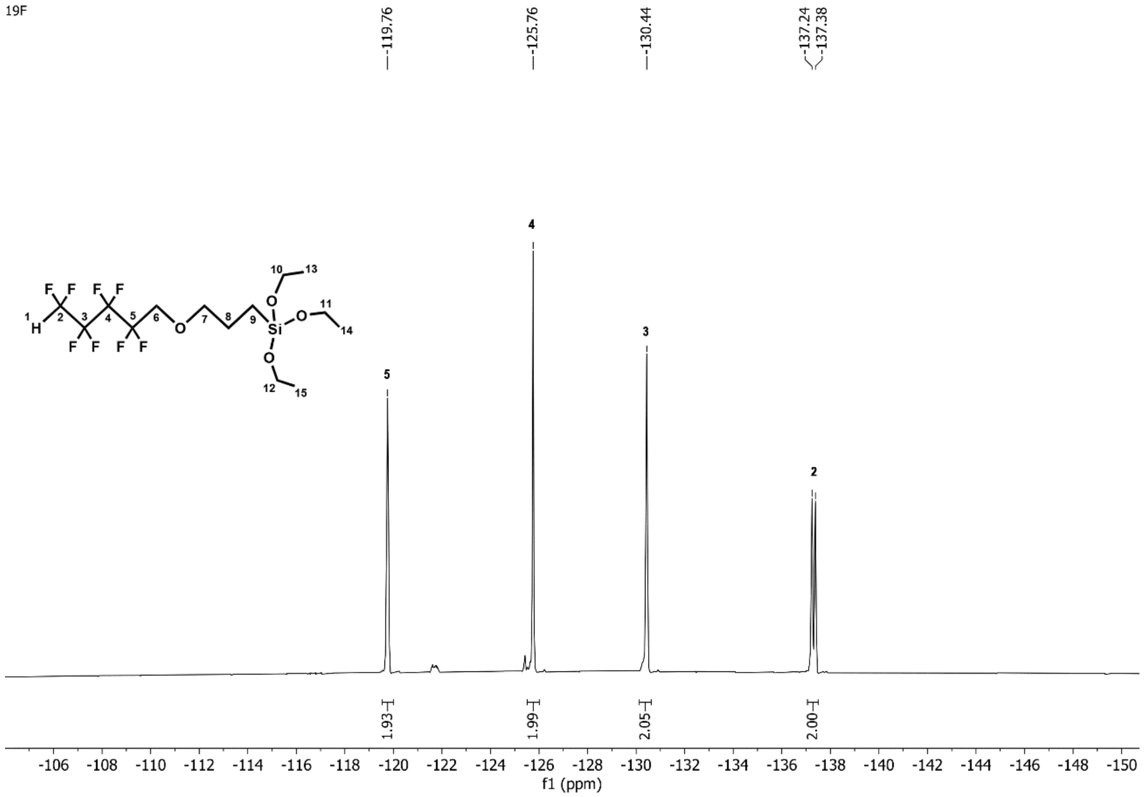

Figure S18. ^{19}F NMR spectrum of triethoxy{3-[(2,2,3,3,4,4,5,5-octafluoropentyl)oxy]propyl}silane **OFTS** in $CDCl_3$.

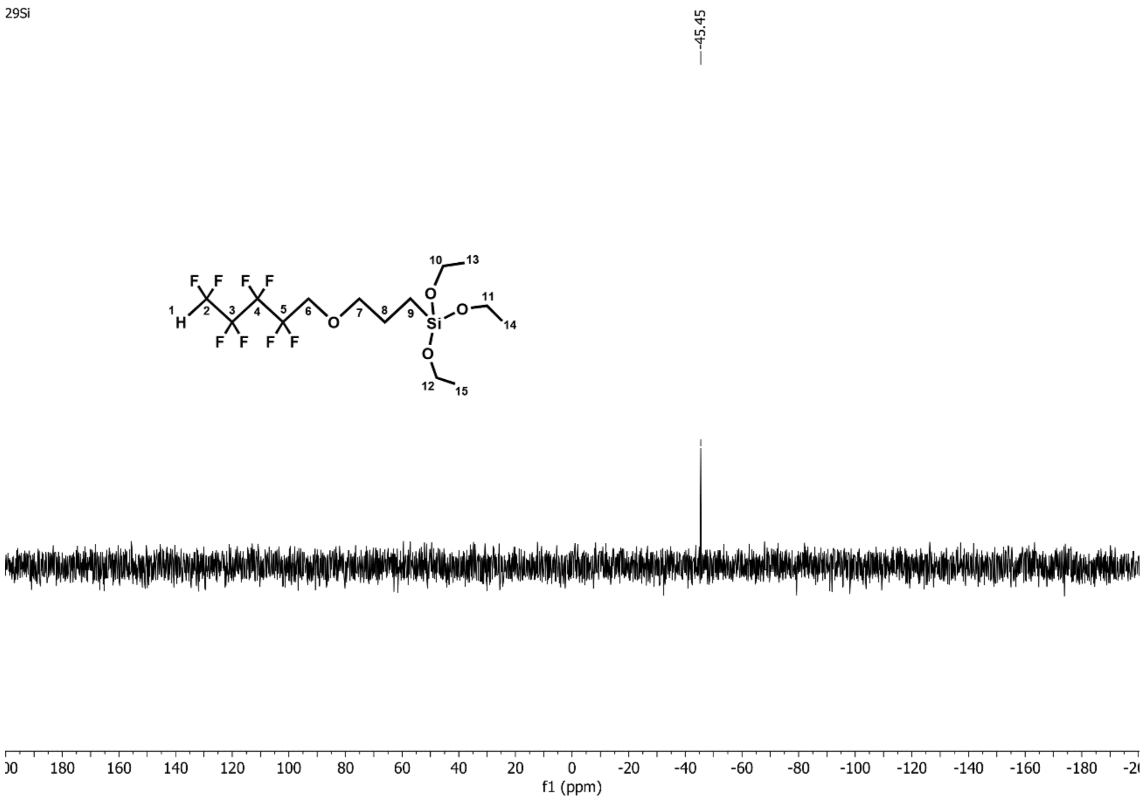

Figure S19. ^{29}Si NMR spectrum of triethoxy{3-[(2,2,3,3,4,4,5,5-octafluoropentyl)oxy]propyl} silane **OFTS** in $CDCl_3$.

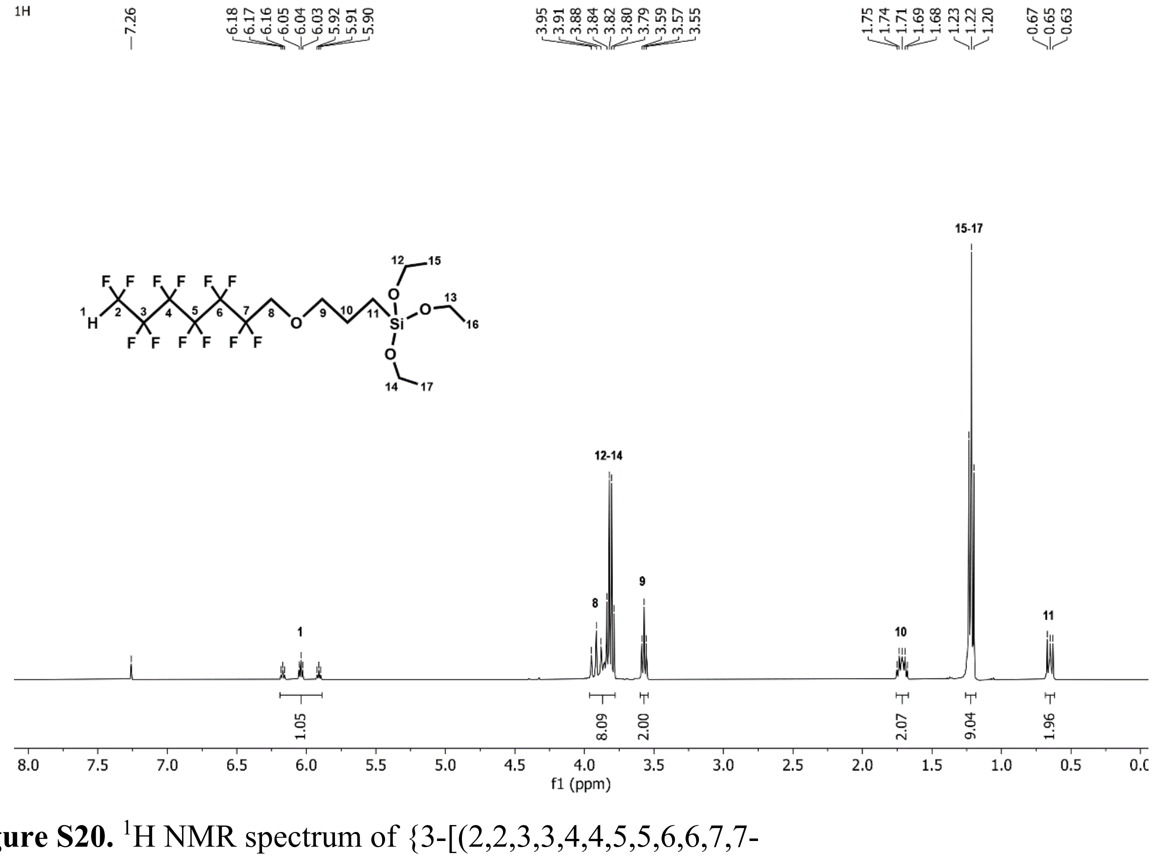

Figure S20. ^{1}H NMR spectrum of {3-[(2,2,3,3,4,4,5,5,6,6,7,7-dodecafluoroheptyl)oxy]propyl}(tri-ethoxy)silane **DFTS** in $CDCl_3$.

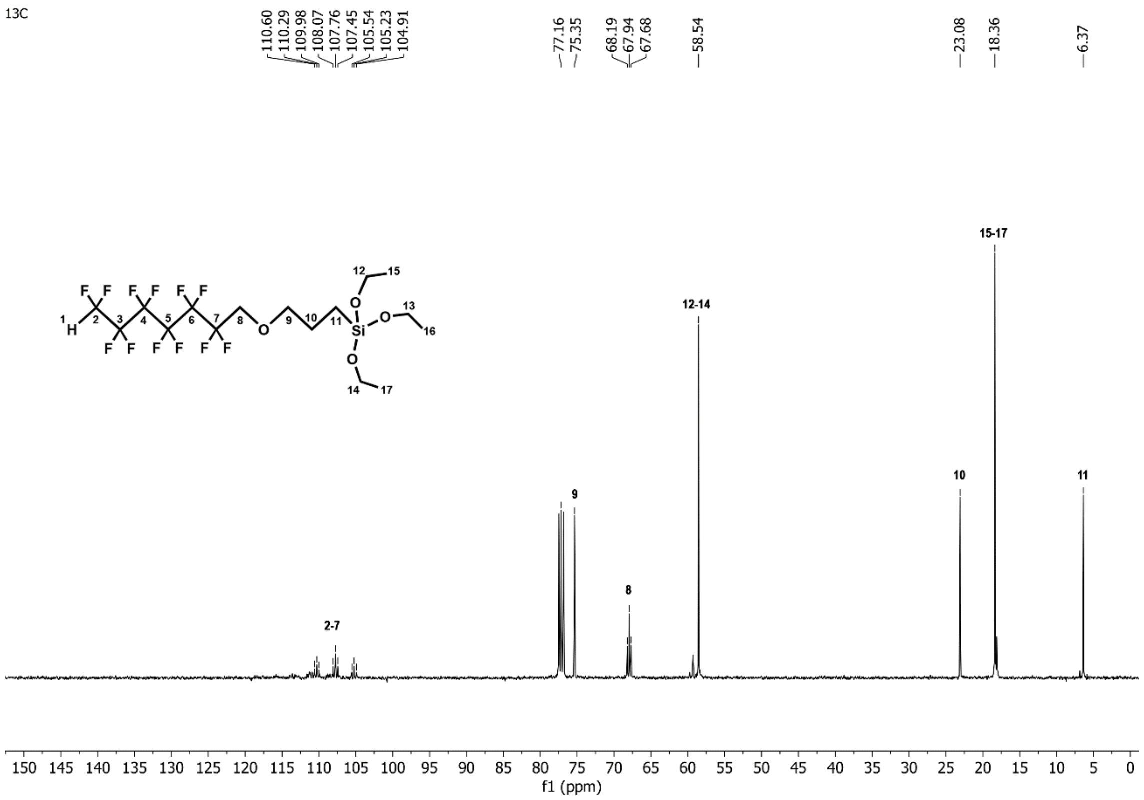

Figure S21. ^{13}C NMR spectrum of {3-[(2,2,3,3,4,4,5,5,6,6,7,7-dodecafluoroheptyl)oxy]propyl}(tri-ethoxy)silane **DFTS** in $CDCl_3$.

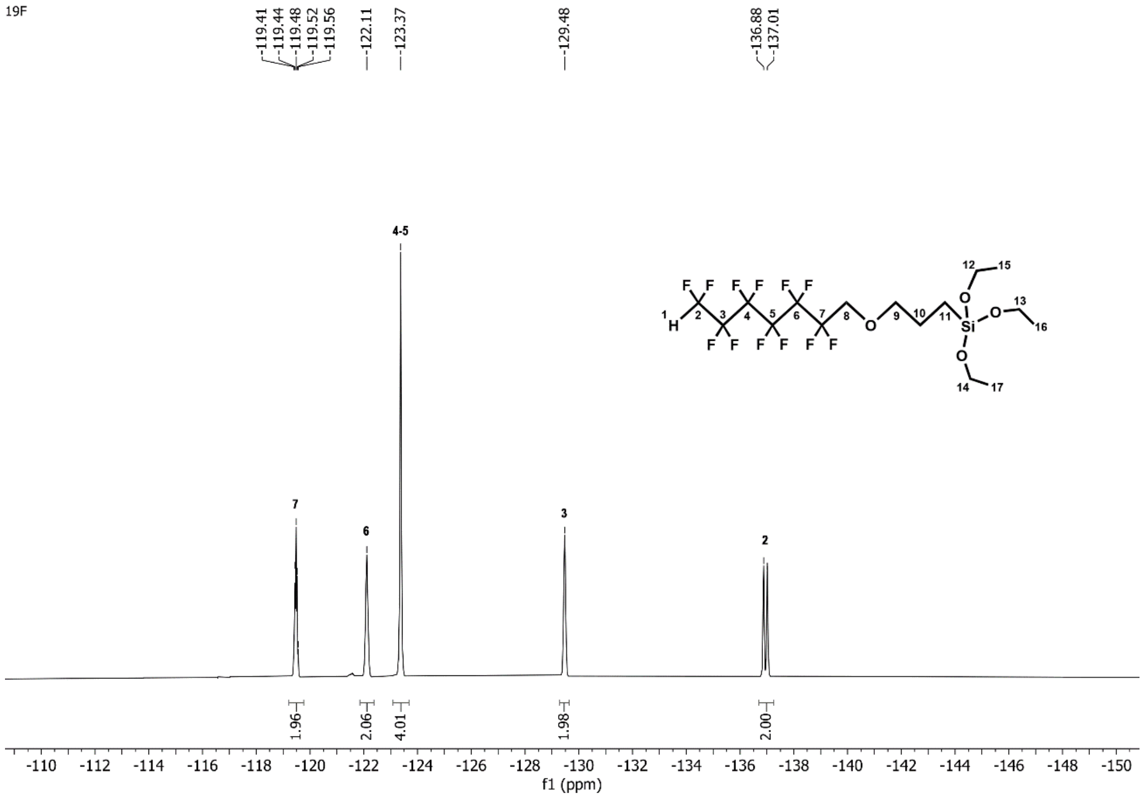

Figure S22. ^{19}F NMR spectrum of {3-[(2,2,3,3,4,4,5,5,6,6,7,7-dodecafluoroheptyl)oxy]propyl}(tri-ethoxy)silane **DFTS** in $CDCl_3$.

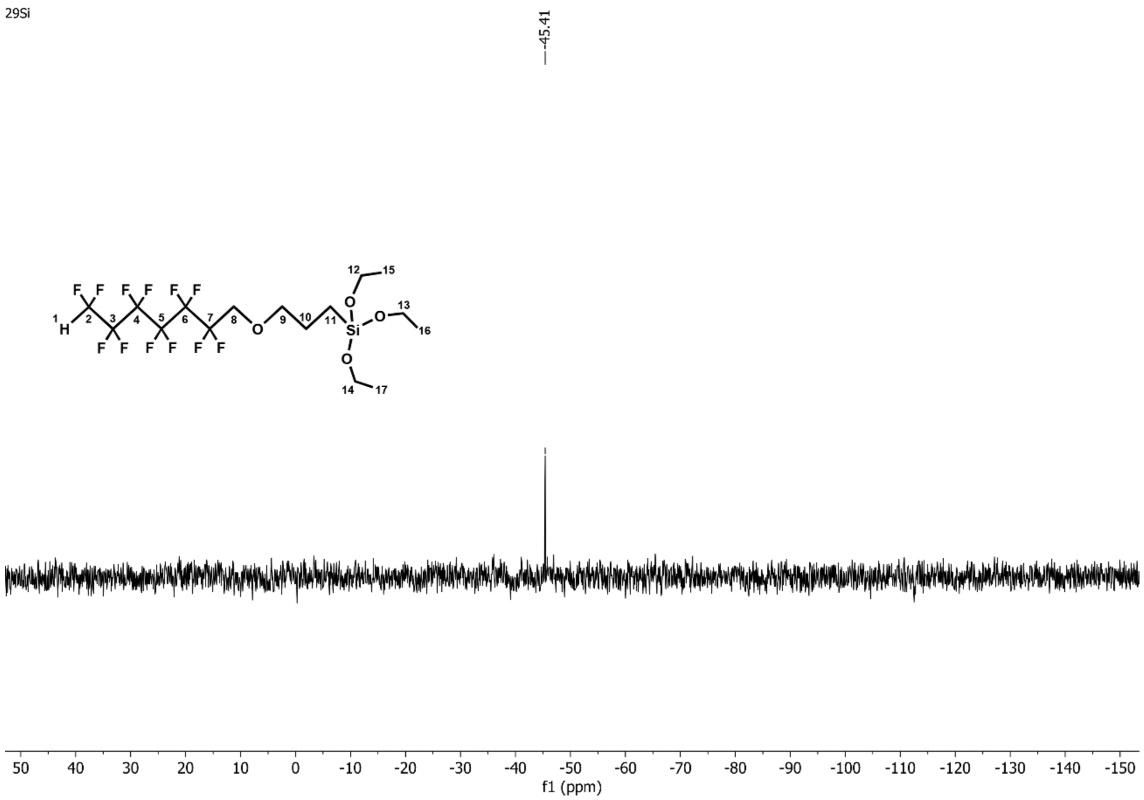

Figure S23. ^{29}Si NMR spectrum of {3-[(2,2,3,3,4,4,5,5,6,6,7,7-dodecafluoroheptyl)oxy]propyl}(tri-ethoxy)silane **DFTS** in $CDCl_3$.

Characterization Data of **TFSQ**

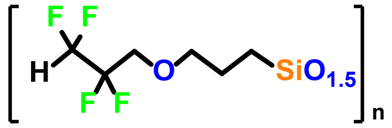

Elemental analysis (%) calculated for $C_6H_9F_4O_{2.5}Si$: C 32.00, H 4.03, F 33.74, Si 12.47; found: C 32.17, H 4.09, F 33.21, Si 12.04.

^{1}H NMR (DMSO-d_6, δ, ppm): 6.59–6.13 (m, 1H, HCF_2), 3.89–3.73 (m, 2H, $CF_2C\underline{H}_2O$), 3.52–3.40 (m, 2H, $OC\underline{H}_2CH_2CH_2Si$), 1.63–1.50 (m, 2H, $OCH_2C\underline{H}_2CH_2Si$), 0.66–0.49 (br m, 2H, $OCH_2CH_2C\underline{H}_2Si$).

^{13}C NMR (DMSO-d_6, δ, ppm): 115.50 ($HCF_2\underline{C}F_2CH_2$, $^1J_{C-F}$ = 263.1 Hz, $^2J_{C-F}$ = 30.6 Hz), 109.31 (tt, HCF_2, $^1J_{C-F}$ = 249.3 Hz, $^2J_{C-F}$ = 33.7 Hz), 73.93–73.25 ($O\underline{C}H_2CH_2CH_2Si$), 66.80 (t, $CF_2\underline{C}H_2O$, $^2J_{C-F}$ = 27.4 Hz), 22.67–22.04 ($OCH_2\underline{C}H_2CH_2Si$), 8.88–7.34 ($OCH_2CH_2\underline{C}H_2Si$).

^{19}F NMR (DMSO-d_6, δ, ppm): -124.90 – -125.18 (2F, $HCF_2C\underline{F}_2CH_2$), -139.61 – -139.82 ($HC\underline{F}_2CF_2CH_2$–).

^{29}Si NMR (DMSO-d_6, δ, ppm): -64.1 – -70.9.

FTIR (solid, ν, cm^{-1}): 2944, 2890 (C–H ν_s and ν_{as}), 1485, 1461, 1415 (C–H δ_{CH}), 1282, 1232, 1205 (C–F), 1110 and 1036 (Si–O–Si), 970 (C–O), 937 (Si-OH), 834 (C–O).

Characterization Data of **OFSQ**

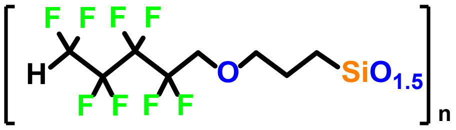

Elemental analysis (%) calculated for $C_8H_9F_8O_{2.5}Si$: C 29.54, H 2.77, F 46.77, Si 8.62; found: C 29.24, H 2.52, F 46.05, Si 8.55.

^{1}H NMR (DMSO-*d*$_6$, δ, ppm): 7.05–6.66 (m, 1H, HCF_2), 4.03–3.84 (m, 2H, $CF_2C\underline{H}_2O$), 3.54–3.42 (m, 2H, $OC\underline{H}_2CH_2CH_2Si$), 1.64–1.51 (m, 2H, $OCH_2C\underline{H}_2CH_2Si$), 0.67–0.49 (br m, 2H, $OCH_2CH_2C\underline{H}_2Si$).

^{13}C NMR (DMSO-*d*$_6$, δ, ppm): 117.78 – 105.41 ($H\underline{C}F_2(\underline{C}F_2)_3CH_2$), 74.09–73.31 ($O\underline{C}H_2CH_2CH_2Si$), 66.53 (t, $CF_2\underline{C}H_2O$, $^2J_{C\text{–}F}$ = 27.4 Hz), 22.76–22.02 ($OCH_2\underline{C}H_2CH_2Si$), 8.84–6.80 ($OCH_2CH_2\underline{C}H_2Si$).

^{19}F NMR (DMSO-*d*$_6$, δ, ppm): -119.40 – -120.23 (2F, $HCF_2CF_2CF_2C\underline{F}_2CH_2$), -125.00 – -125.64 (2F, $HCF_2CF_2C\underline{F}_2CF_2CH_2$–), -129.82 – -130.13 (2F, $HCF_2C\underline{F}_2CF_2CF_2CH_2$–), -138.30 – -138.70 (2F, $HC\underline{F}_2CF_2CF_2CF_2CH_2$–).

^{29}Si NMR (DMSO-*d*$_6$, δ, ppm): -64.37 – -71.60.

FTIR (solid, ν, cm^{-1}): 2937, 2884 (C–H ν_s and ν_{as}), 1485, 1467, 1418 (C–H δ_{CH}), 1285, 1236, 1206 (C–F), 1100 and 1053 (Si–O–Si), 977 (C–O), 944 (Si-OH), 840 (C–O).

Characterization Data of **DFSQ**

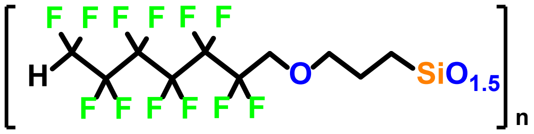

Elemental analysis (%) calculated for $C_{10}H_9F_{12}O_{2.5}Si$: C 28.24, H 2.12, F 53.65, Si 6.59; found: C 28.21, H 2.06, F 53.20, Si 6.36.

^{1}H NMR ($(CD_3)_2CO$, δ, ppm): 6.93–6.57 (m, 1H, HCF_2), 4.11–3.98 (m, 2H, $CF_2C\underline{H}_2O$), 3.70–3.60 (m, 2H, $OC\underline{H}_2CH_2CH_2Si$), 1.85–1.70 (m, 2H, $OCH_2C\underline{H}_2CH_2Si$), 0.84–0.68 (br m, 2H, $OCH_2CH_2C\underline{H}_2Si$).

^{13}C NMR ($(CD_3)_2CO$, δ, ppm): 119.47–106.48 ($H\underline{C}F_2(\underline{C}F_2)_5CH_2$), 76.08–75.22 ($O\underline{C}H_2CH_2CH_2Si$), 68.07 (t, $CF_2\underline{C}H_2O$, $^2J_{C\text{–}F}$ = 27.4 Hz), 24.23–23.38 ($OCH_2\underline{C}H_2CH_2Si$), 10.00–8.29 ($OCH_2CH_2\underline{C}H_2Si$).

^{19}F NMR ($(CD_3)_2CO$, δ, ppm): -120.04 – -120.52 (2F, $HCF_2CF_2CF_2CF_2CF_2C\underline{F}_2CH_2$), -122.64 – -122.88 (2F, $HCF_2CF_2CF_2CF_2C\underline{F}_2CF_2CH_2$–), -123.78 – -124.10 (4F, $HCF_2CF_2C\underline{F}_2C\underline{F}_2CF_2CF_2CH_2$–), -130.02 – -130.25 (2F, $HCF_2C\underline{F}_2CF_2CF_2CF_2CF_2CH_2$–), -138.78 – -139.09 (2F, $HC\underline{F}_2CF_2CF_2CF_2CF_2CF_2CH_2$–).

^{29}Si NMR ($(CD_3)_2CO$, δ, ppm): -63.62 – -71.30.

FTIR (solid, ν, cm^{-1}): 2937, 2880 (C–H ν_s and ν_{as}), 1478, 1458, 1435, 1411 (C–H δ_{CH}), 1260, 1197, 1167 (C–F), 1120 and 1050 (Si–O–Si), 941 (Si-OH), 831 (C–O).

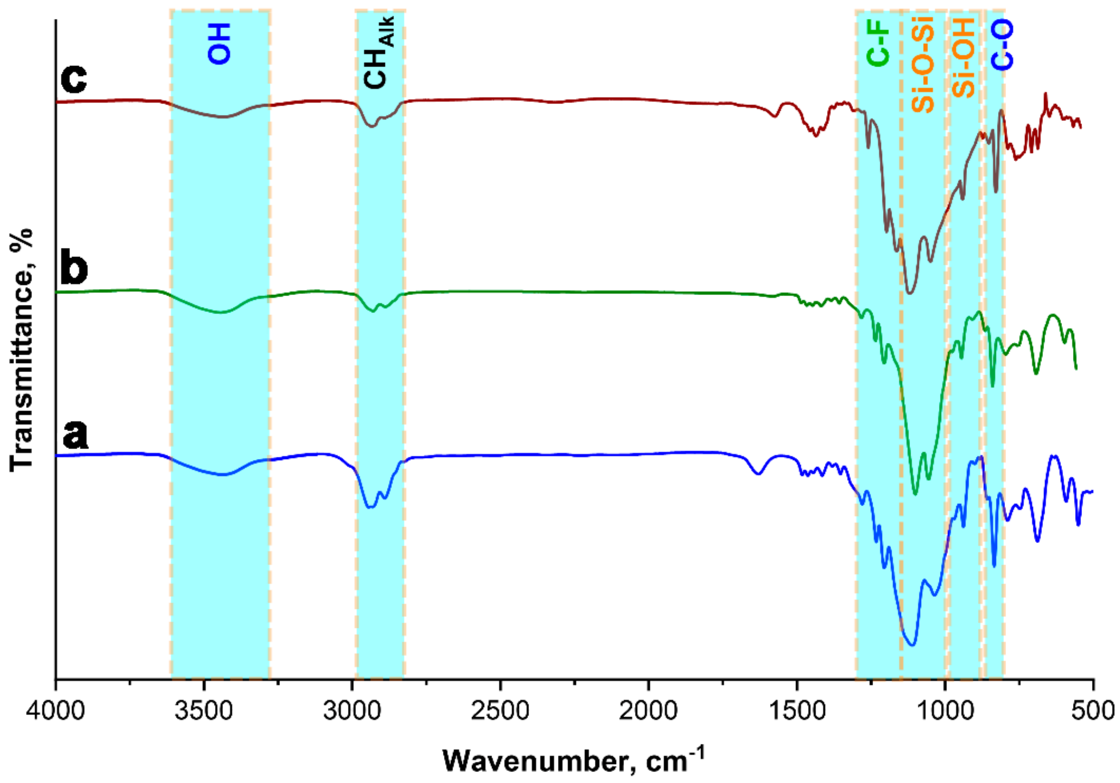

Figure S24. FTIR spectra of {[3-(2,2,3,3-tetrafluoropropoxy)propyl]silsesquioxane **TFSQ** (a), {3-[(2,2,3,3,4,4,5,5-octafluoropentyl)oxy]propyl}silsesquioxane **OFSQ** (b), and {3-[(2,2,3,3,4,4,5,5,6,6,7,7-dodecafluoroheptyl)oxy]propyl} silsesquioxane **DFSQ** (c).

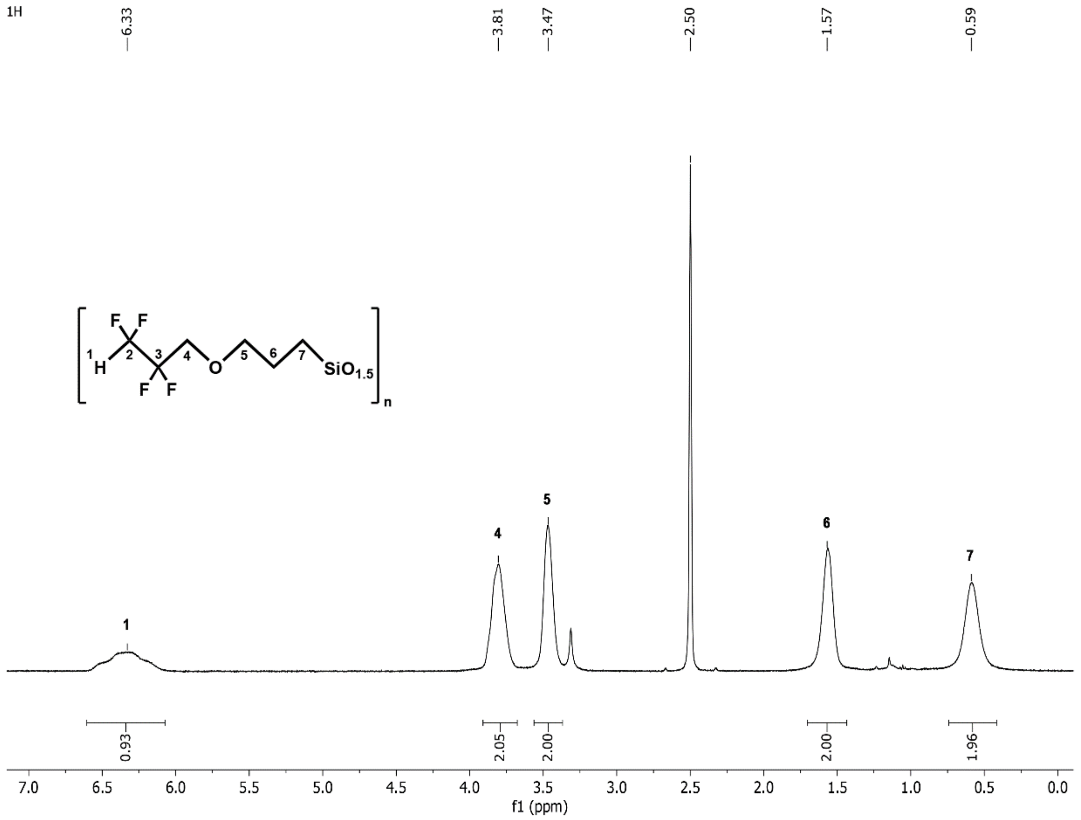

Figure S25. ^{1}H NMR spectrum of {[3-(2,2,3,3-tetrafluoropropoxy)propyl]silsesquioxane **TFSQ** in DMSO-d_6.

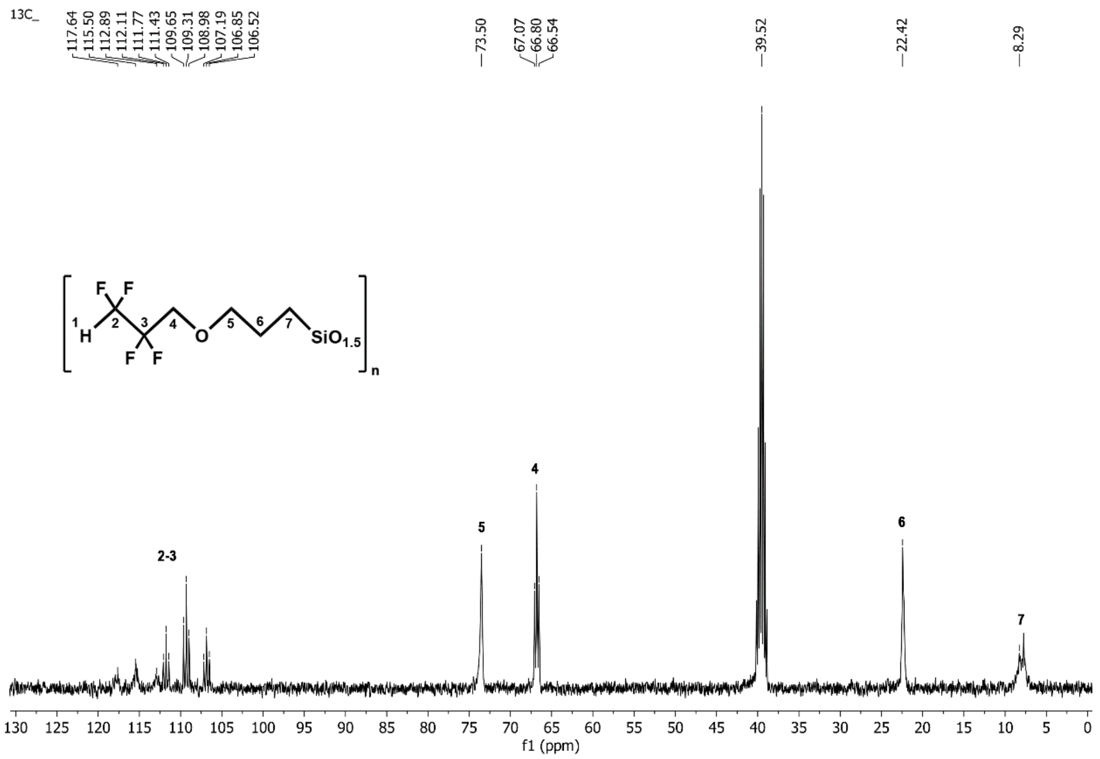

Figure S26. ^{13}C NMR spectrum of {[3-(2,2,3,3-tetrafluoropropoxy)propyl]silsesquioxane **TFSQ** in DMSO-d_6.

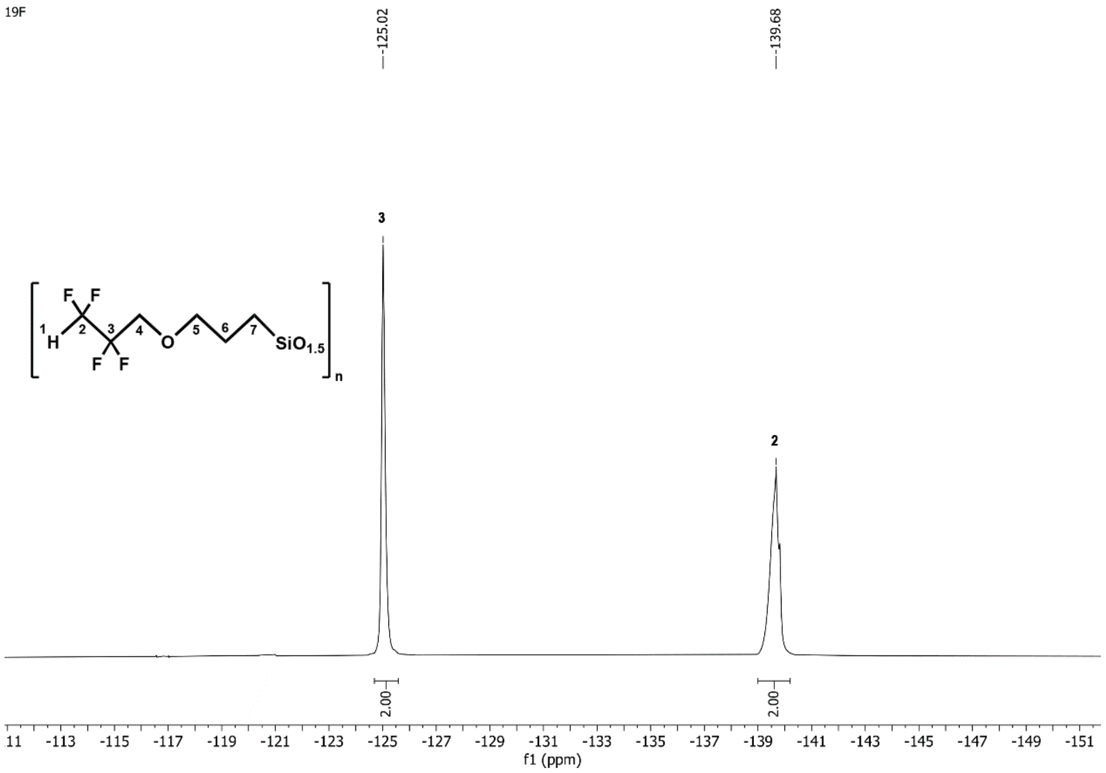

Figure S27. ^{19}F NMR spectrum of {[3-(2,2,3,3-tetrafluoropropoxy)propyl]silsesquioxane **TFSQ** in DMSO-d_6.

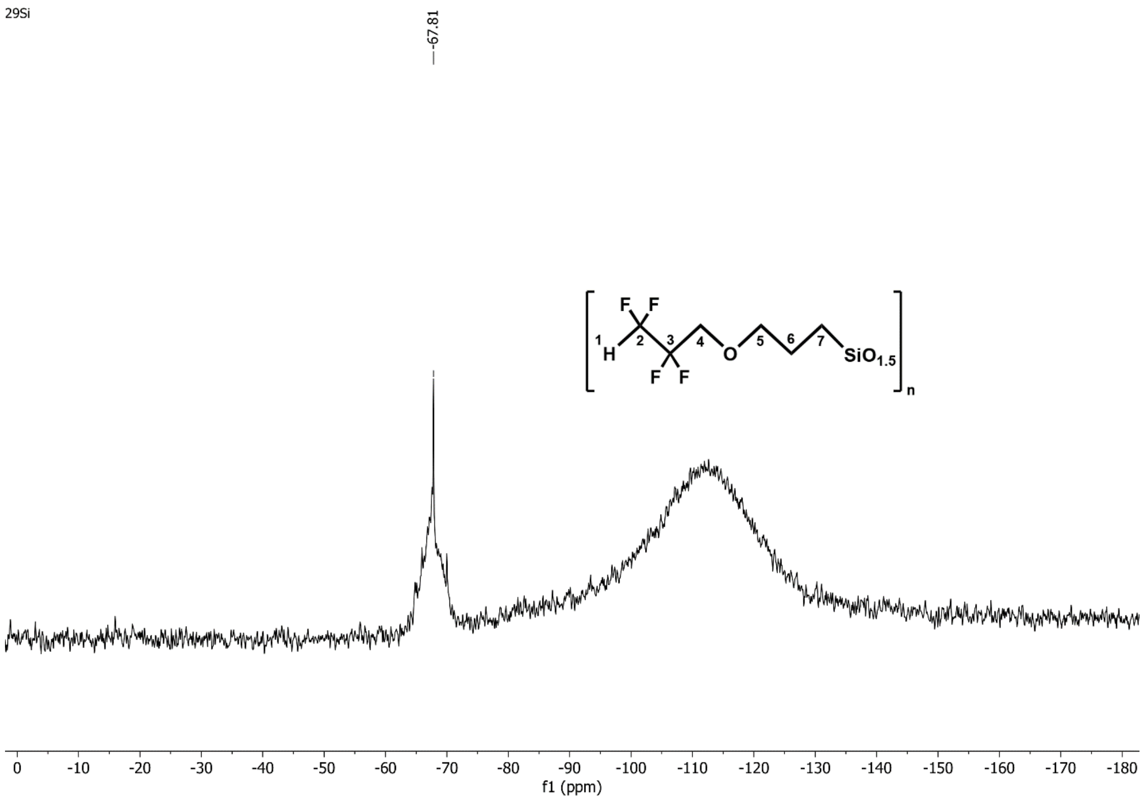

Figure S28. ^{29}Si NMR spectrum of {[3-(2,2,3,3-tetrafluoropropoxy)propyl]silsesquioxane **TFSQ** in DMSO-d_6.

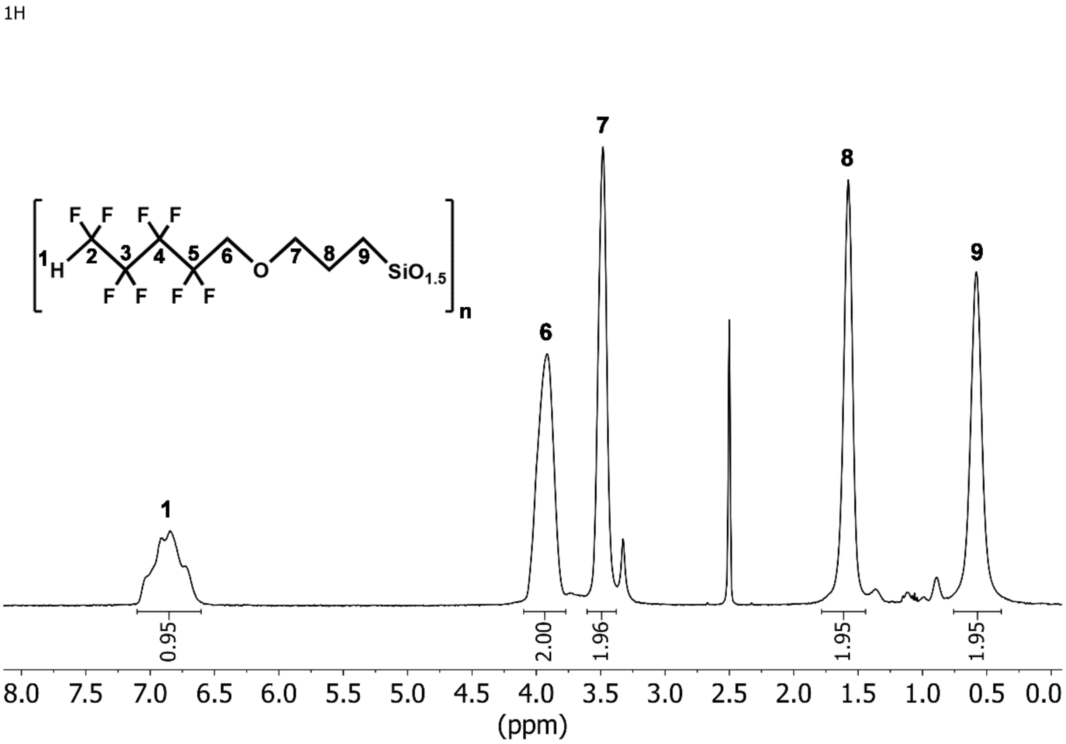

Figure S29. ^{1}H NMR spectrum of {3-[(2,2,3,3,4,4,5,5-octafluoropentyl)oxy]propyl}silsesquioxane **OFSQ** in DMSO-d_6.

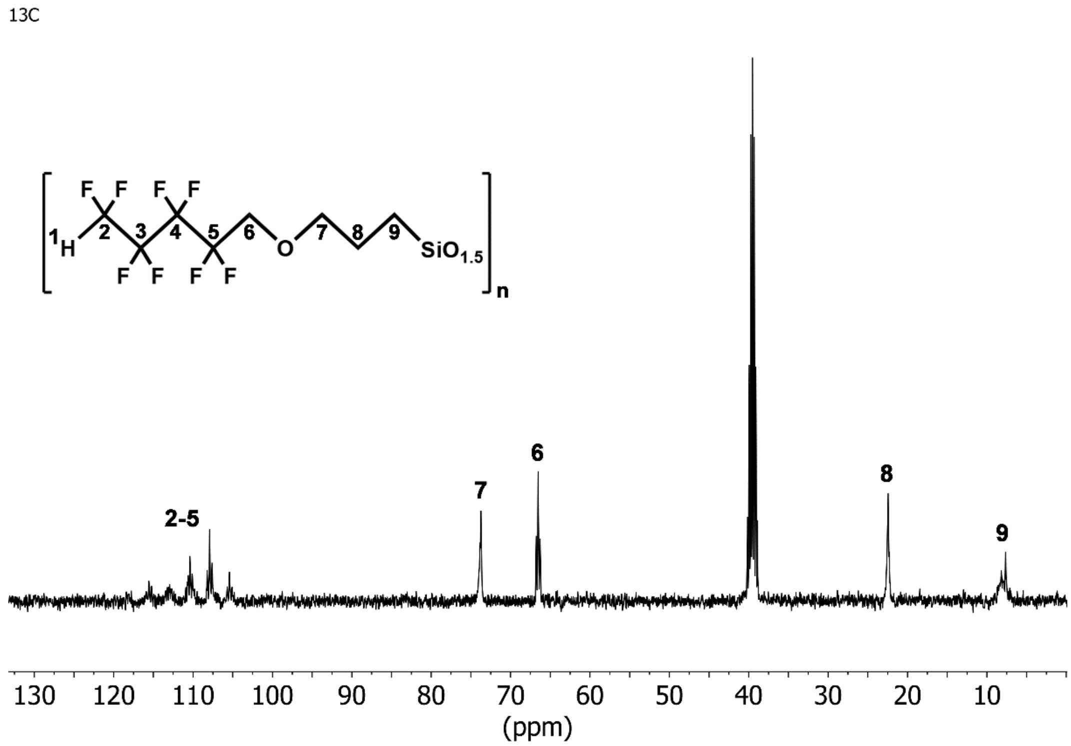

Figure S30. ^{13}C NMR spectrum of {3-[(2,2,3,3,4,4,5,5-octafluoropentyl)oxy]propyl}silsesquioxane **OFSQ** in DMSO-d_6.

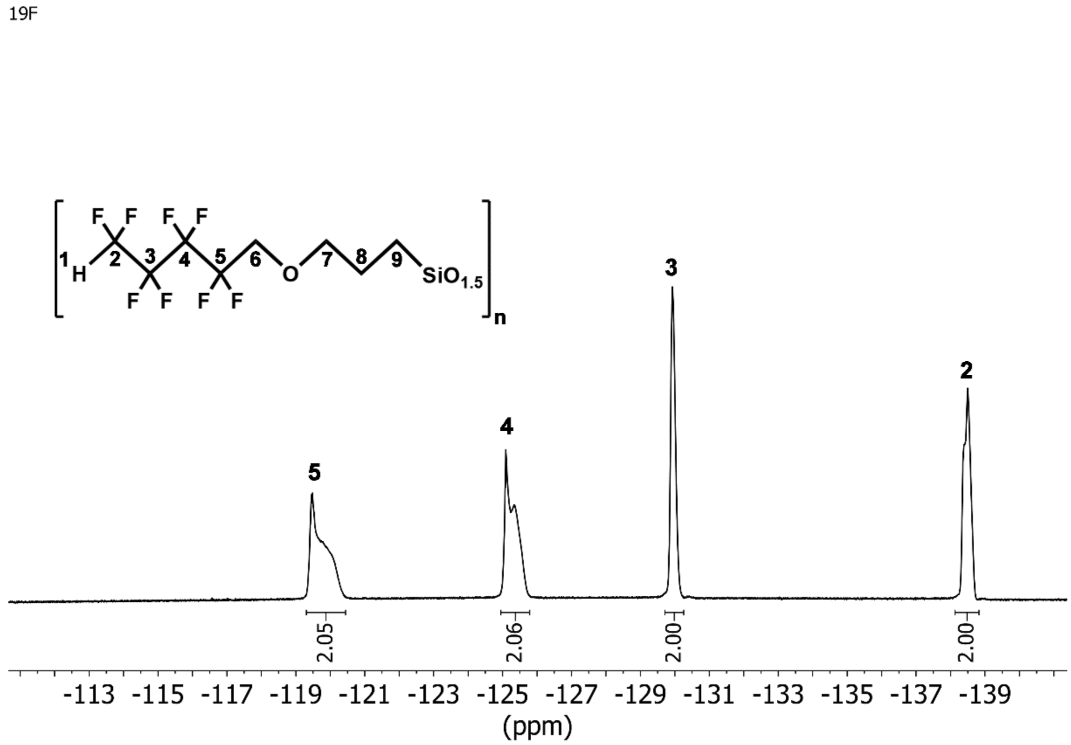

Figure S31. ^{19}F NMR spectrum of {3-[(2,2,3,3,4,4,5,5-octafluoropentyl)oxy]propyl}silsesquioxane **OFSQ** in DMSO-d_6.

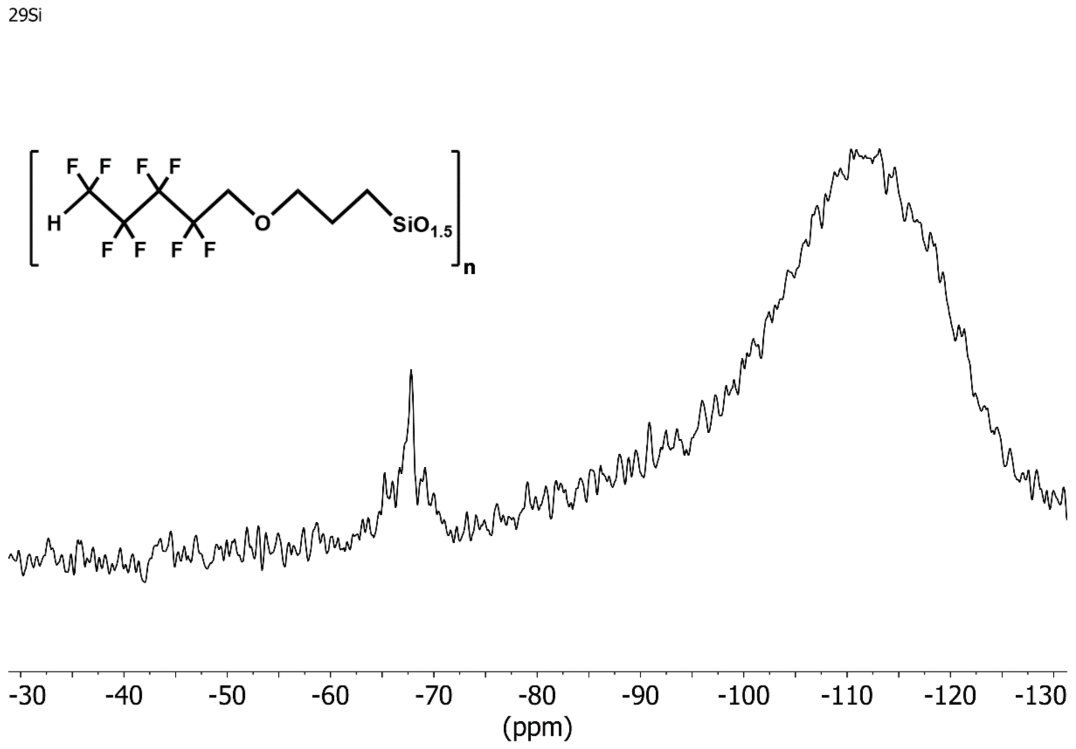

Figure S32. ^{29}Si NMR spectrum of {3-[(2,2,3,3,4,4,5,5-octafluoropentyl)oxy]propyl} silsesquioxane **OFSQ** in DMSO-d_6.

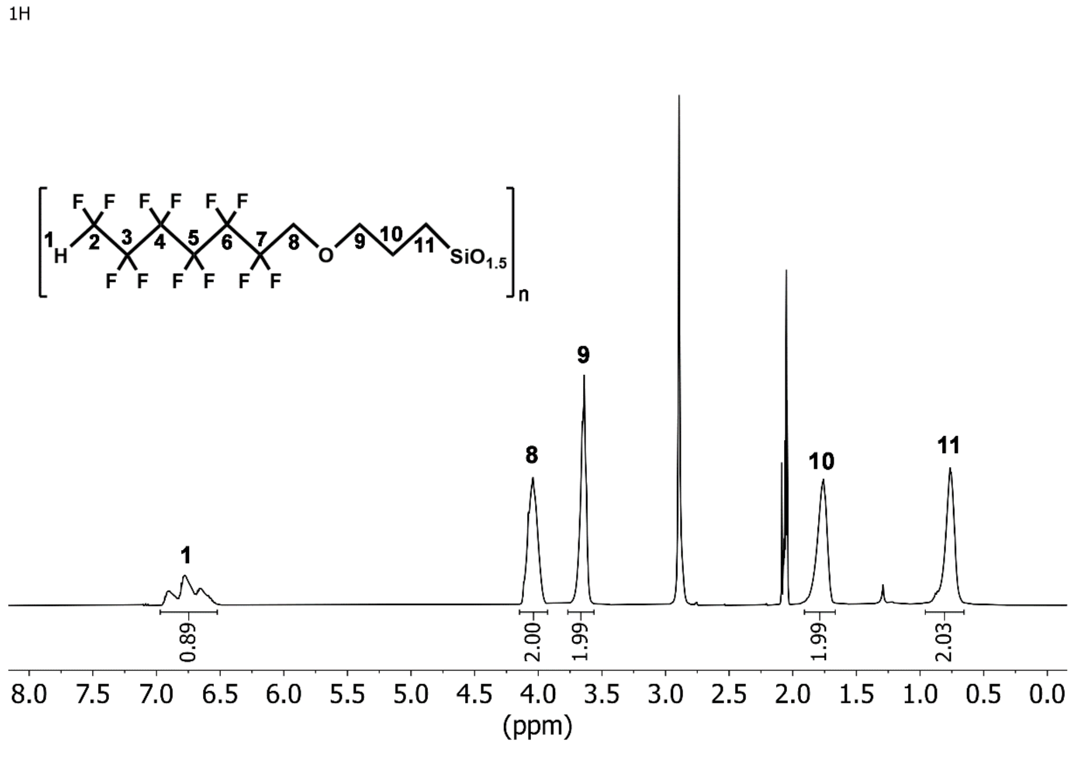

Figure S33. ^{1}H NMR spectrum of {3-[(2,2,3,3,4,4,5,5,6,6,7,7-dodecafluoroheptyl)oxy]propyl} silsesquioxane **DFSQ** in $(CD_3)_2CO$.

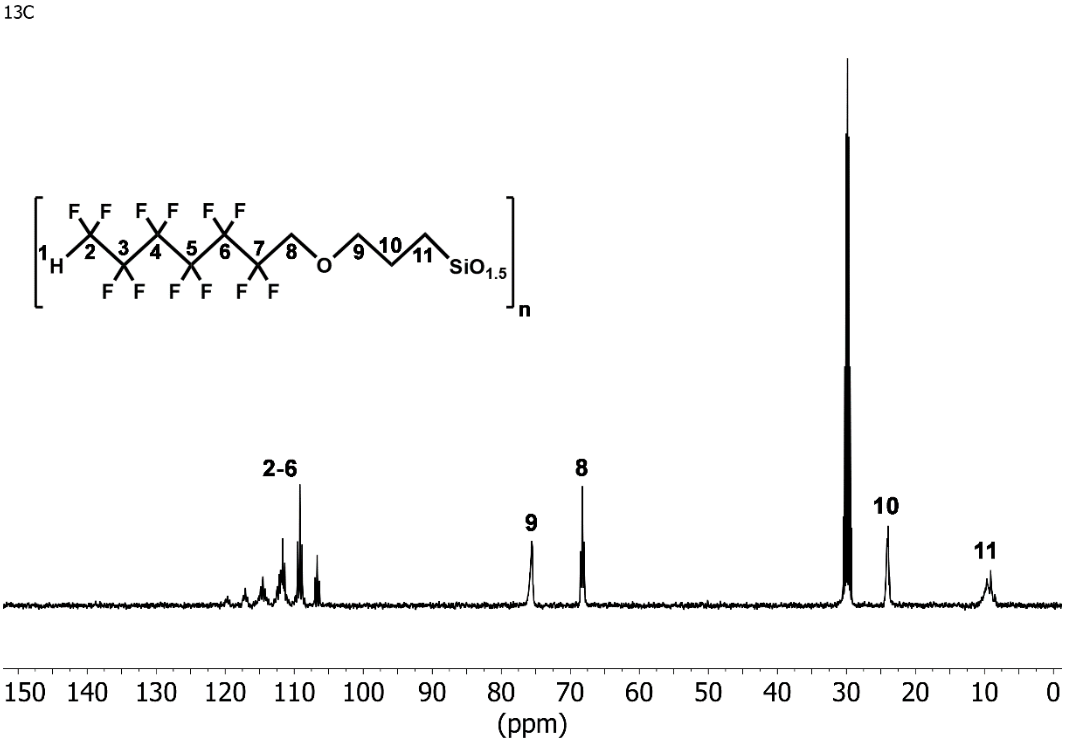

Figure S34. ^{13}C NMR spectrum of {3-[(2,2,3,3,4,4,5,5,6,6,7,7-dodecafluoroheptyl)oxy]propyl} silsesquioxane **DFSQ** in $(CD_3)_2CO$.

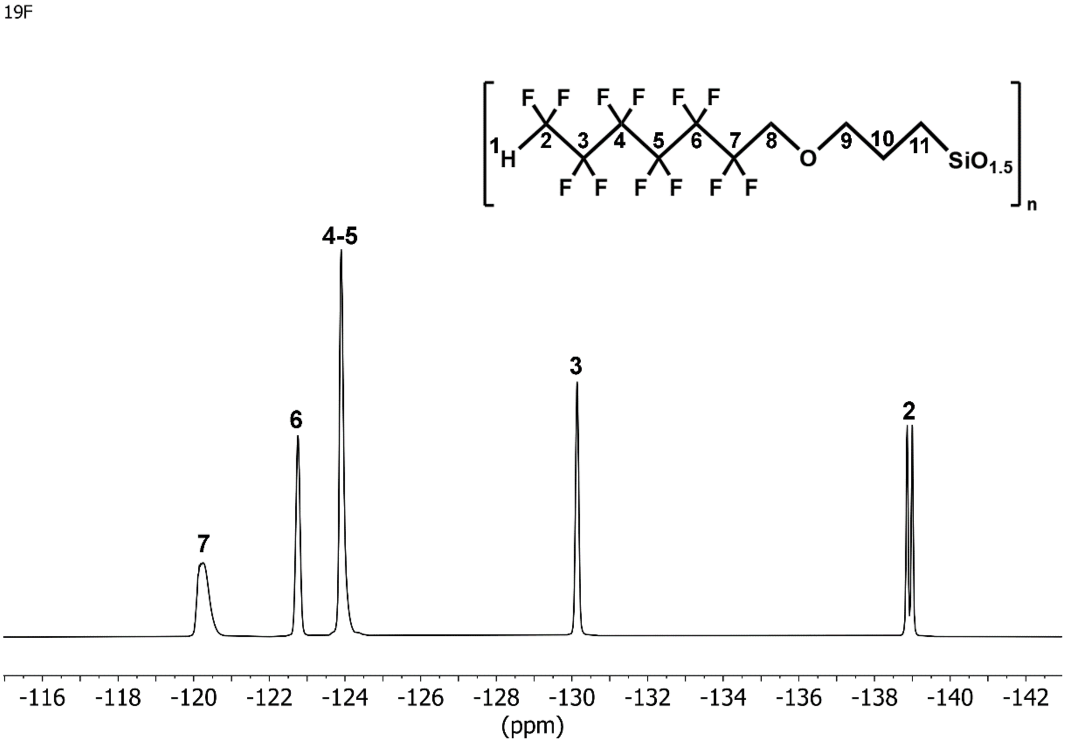

Figure S35. ^{19}F NMR spectrum of {3-[(2,2,3,3,4,4,5,5,6,6,7,7-dodecafluoroheptyl)oxy]propyl} silsesquioxane **DFSQ** in $(CD_3)_2CO$.

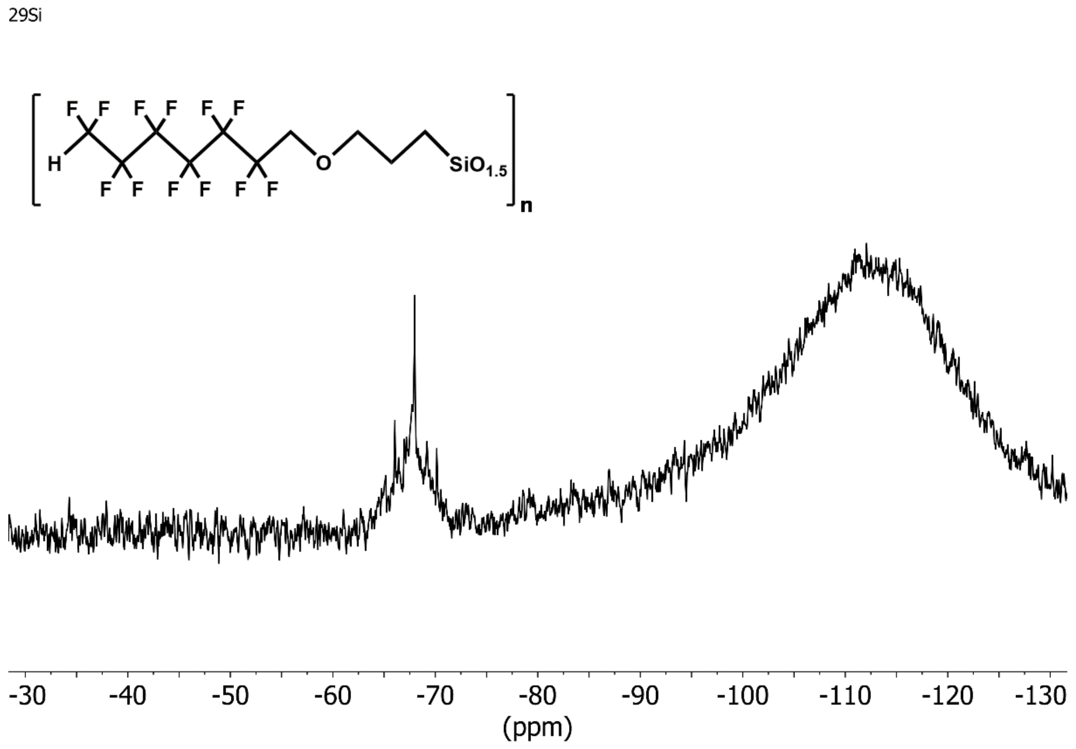

Figure S36. ^{29}Si NMR spectrum of {3-[(2,2,3,3,4,4,5,5,6,6,7,7-dodecafluoroheptyl)oxy]propyl} silsesquioxane **DFSQ** in $(CD_3)_2CO$.

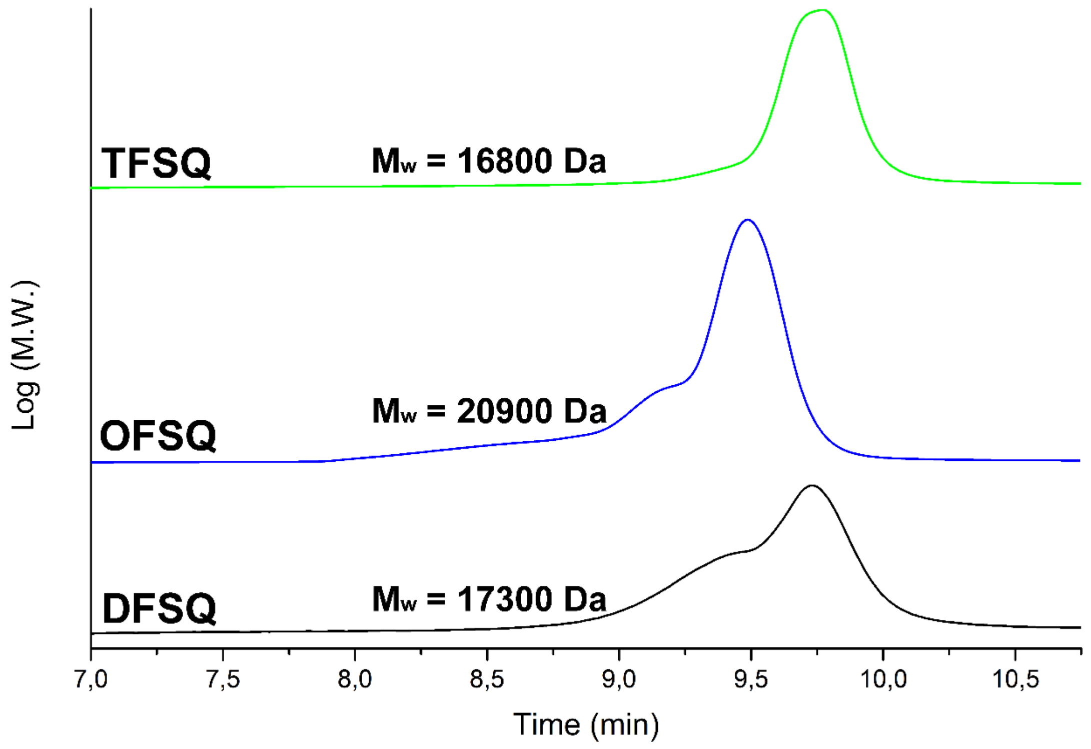

Figure S37. The gel permeation chromatography curves of **TFSQ**, **OFSQ**, and **DFSQ**.

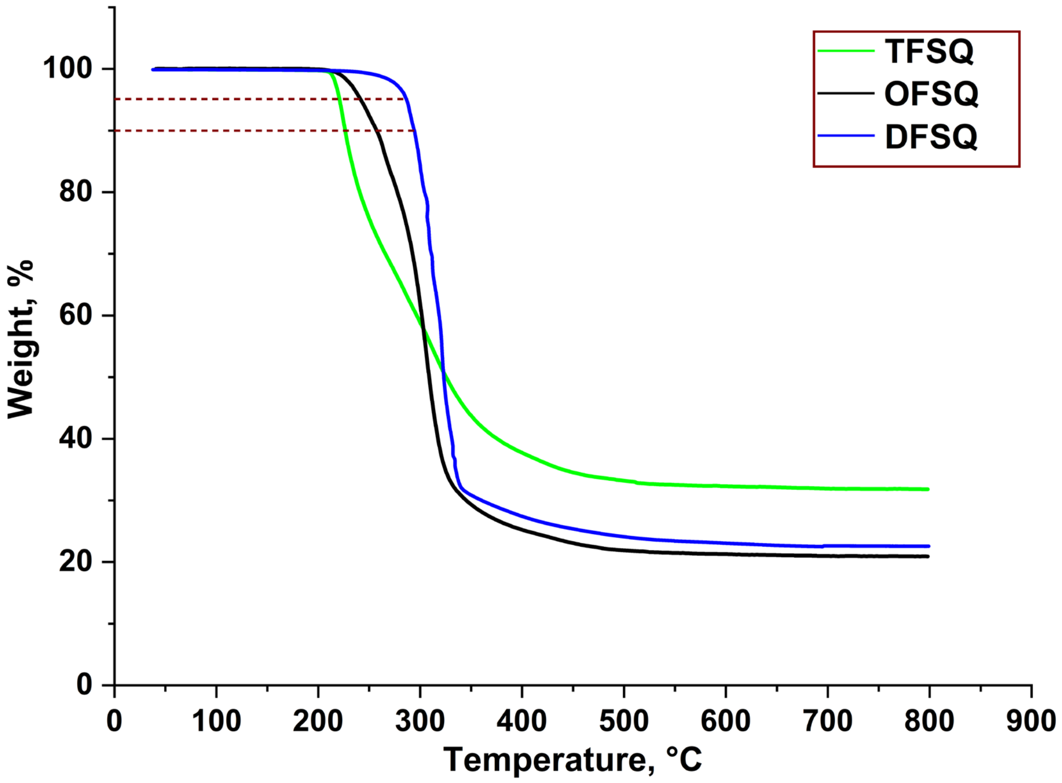

Figure S38. TGA curves of **TFSQ**, **OFSQ**, and **DFSQ**.

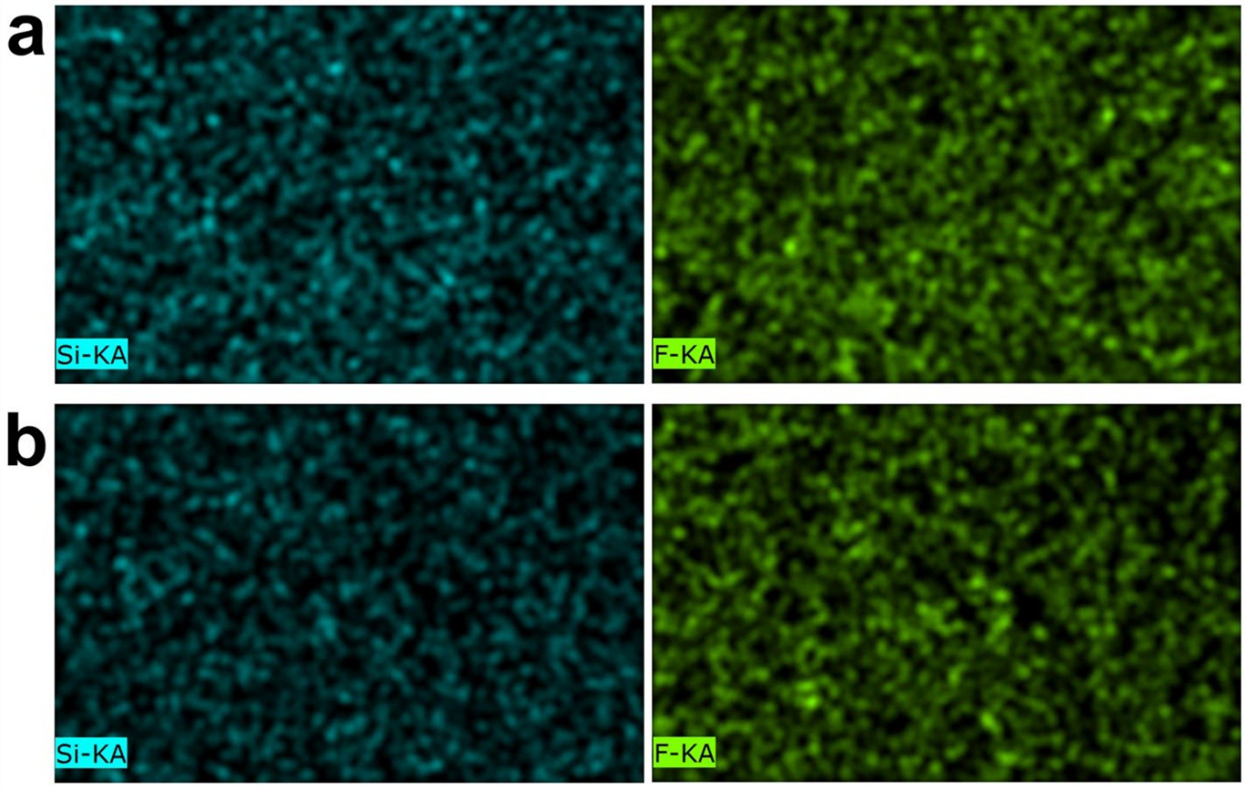

Figure S39. EDS mapping of Si and F elements on the surface of **TFSQ$_{5\%}$** (**a**) and **OFSQ$_{5\%}$** (**b**).
